# Associations Between Physical Characteristics and Golf Clubhead Speed: A Systematic Review with Meta-Analysis

**DOI:** 10.1007/s40279-024-02004-5

**Published:** 2024-02-29

**Authors:** Alex Brennan, Andrew Murray, Margo Mountjoy, John Hellstrom, Dan Coughlan, Jack Wells, Simon Brearley, Alex Ehlert, Paul Jarvis, Anthony Turner, Chris Bishop

**Affiliations:** 1 Faculty of Science and Technology, London Sport Institute, Middlesex University, London, UK; 2 Medical and Scientific Department, The R&A, St Andrews, UK; 3 European Tour Health and Performance Institute, European Tour Group, Virginia Water, UK; 4 Health and Performance Institute, Ladies European Tour, Denham, UK; 5 Department of Family Medicine, McMaster University, Hamilton, Canada; 6 International Golf Federation, Lausanne, Switzerland; 7 International Olympic Committee Games Group, Lausanne, Switzerland; 8 Swedish Golf Federation, Stockholm, Sweden; 9 England Golf, Woodhall Spa, Lincolnshire, UK; 10 Cambridge Centre for Sport & Exercise Sciences, Anglia Ruskin University, Cambridge, UK; 11 Independent Researcher, Knightdale, NC USA

## Abstract

**Background:**

Historically, golf does not have a strong tradition of fitness testing and physical training. However, in recent years, both players and practitioners have started to recognise the value of a fitter and healthier body, owing to its potential positive impacts on performance, namely clubhead speed (CHS).

**Objective:**

The aim of this meta-analysis was to examine the associations between CHS (as measured using a driver) and a variety of physical characteristics.

**Methods:**

A systematic literature search with meta-analysis was conducted using Medline, SPORTDiscus, CINAHL and PubMed databases. Inclusion criteria required studies to have (1) determined the association between physical characteristics assessed in at least one physical test and CHS, (2) included golfers of any skill level but they had to be free from injury and (3) been peer-reviewed and published in the English language. Methodological quality was assessed using a modified version of the Downs and Black Quality Index tool and heterogeneity assessed via the *Q* statistic and *I*^2^. To provide summary effects for each of the physical characteristics and their associations with CHS, a random effects model was used where *z*-transformed *r* values (i.e. *z*_*r*_) were computed to enable effect size pooling within the meta-analysis.

**Results:**

Of the 3039 studies initially identified, 20 were included in the final analysis. CHS was significantly associated with lower body strength (*z*_*r*_ = 0.47 [95% confidence intervals {CI} 0.24–0.69]), upper body strength (*z*_*r*_ = 0.48 [95% CI 0.28–0.68]), jump displacement (*z*_*r*_ = 0.53 [95% CI 0.28–0.78]), jump impulse (*z*_*r*_ = 0.82 [95% CI 0.63–1.02]), jumping peak power (*z*_*r*_ = 0.66 [95% CI 0.53–0.79]), upper body explosive strength (*z*_*r*_ = 0.67 [95% CI 0.53–0.80]), anthropometry (*z*_*r*_ = 0.43 [95% CI 0.29–0.58]) and muscle capacity (*z*_*r*_ = 0.17 [95% CI 0.04–0.31]), but not flexibility (*z*_*r*_ = − 0.04 [95% CI − 0.33 to 0.26]) or balance (*z*_*r*_ =  − 0.06 [95% CI − 0.46 to 0.34]).

**Conclusions:**

The findings from this meta-analysis highlight a range of physical characteristics are associated with CHS. Whilst significant associations ranged from trivial to large, noteworthy information is that jump impulse produced the strongest association, upper body explosive strength showed noticeably larger associations than upper body strength, and flexibility was not significant. These findings can be used to ensure practitioners prioritise appropriate fitness testing protocols for golfers.

## Key Points

**Table:** 

Clubhead speed (CHS) is one of the most important factors for golfers when aiming to optimise distance from tee shots, as it helps to offer an advantage over their competitors.
From empirical investigations, numerous physical characteristics have been shown to be associated with CHS.
Specifically, explosive force production in the lower body (as measured by jumping) elicits stronger associations than maximal lower body strength.
Similarly, upper body explosive strength appears to be of greater importance than upper body strength, most likely because it better replicates the time constraints that the upper body has to produce force during the swing.
Contrary to popular historical belief, flexibility was one of two physical characteristics not significantly associated with CHS. This is likely because a wide range of movement strategies are available for golfers during the swing, enabling a variety of movement solutions to be used, to achieve the desired swing technique.

## Introduction

Golf is a globally played sport with an estimated 66 million people playing worldwide at the turn of the twenty-first century [1]. With the rise in participation rates, there is a growing interest in performance science for the sport, which has been highlighted by the recent increase in studies focusing on performance, medicine and health [2–5]. Given the wide array of abilities across these millions of golfers, authors have often looked to categorise players based on skill. For example, the International Golf Federation consensus statement on reporting and recording of injuries in golf suggested three descriptive classifications: (1) *elite* (professional players competing on tour or amateurs competing in international or national amateur championships); (2) *sub-elite* (Professional Golfers’ Association (PGA) teaching professionals, amateurs competing in regional, county and state tournaments, or with a handicap ≤ 5); and (3) *recreational* (handicap > 5) [6]. These classifications are determined by skill-related parameters (i.e. performance metrics that combine all aspects of a golfer’s performance). For example, handicap index is a score given during recreational golf, which provides golfers with a shot allowance relative to their skill level. In addition, handicap index also considers factors relating to the difficulty of the course, such as length of each hole, course rating and slope. In contrast, a ‘gross’ score (no adjustment on the final scoring) is given to determine performance within professional and high-level amateur competitions. Even though skill-related parameters differentiate across competitive abilities, the aim of golf remains the same: to complete each hole in as few shots as possible and ultimately achieve the lowest score attainable.

From a physical preparation standpoint, research has increased significantly in recent years, with training programmes now frequently used to enhance swing performance [7]. The ability to achieve maximal golf shot distance (largely on par 4 and 5 holes) is of great importance, as it is associated with lower scores, assuming that accuracy can be maintained [8]. An essential underpinning factor for achieving maximal shot distance is clubhead speed (CHS), which, if increased, will result in subsequent increases in other critical parameters such as ball speed, carry distance and total distance, when all other variables are held constant [9]. Consequently, CHS is a metric consistently utilised in the field of golf research as it reflects positive performance in the sport [10]. However, both distance and accuracy determine the outcome of any given shot, with a plethora of launch characteristics (e.g. ball speed, spin rate, launch angle) and impact factors (e.g. impact location, club path, face angle) concurrently responsible for where a shot ends up [11]. Despite the importance of these additional metrics, almost all research to date linking golf performance to physical characteristics has been conducted using CHS as the key performance indicator, with substantially less focus on other shot metrics or skill-related parameters. Therefore, understanding the association between physical capacities and CHS provides vital information on which physical qualities need prioritising for the testing and preparation of players.

Various physical characteristics have been suggested to contribute to golf performance and CHS [12, 13]. For example, previous research has reported that the one repetition maximum (1RM) back squat had a large correlation with CHS (*r* = 0.54; *p* < 0.001) in thirty-three elite male golfers [14]. Further, the same study also reported a large correlation between countermovement jump (CMJ) height and CHS (*r* = 0.61; *p* < 0.001) [14]. Further to this, additional characteristics, such as flexibility, have also been shown to be potentially important in golf, with large correlations between CHS and seated trunk rotation in the clockwise and anti-clockwise directions (*r* = 0.52; *p* < 0.001; *r* = 0.711; *p* < 0.001, respectively) [15]. Thus, the identification of key physical attributes associated with CHS allows practitioners to test and monitor the strengths and weaknesses of golfers, providing more appropriate suggestions on how to individualise their physical training [12, 16], and was the primary aim of this meta-analysis. Whilst this has already been reported in previous golf research [13], the associated *r* values were lifted directly from each study. In contrast, the present investigation undertook more advanced statistical analyses, enabling pooled effect sizes to be conducted and the same level of analysis provided for each empirical study that met our inclusion criteria.

## Methods

### Study Design and Literature Search Methodology

**Fig. 1:**
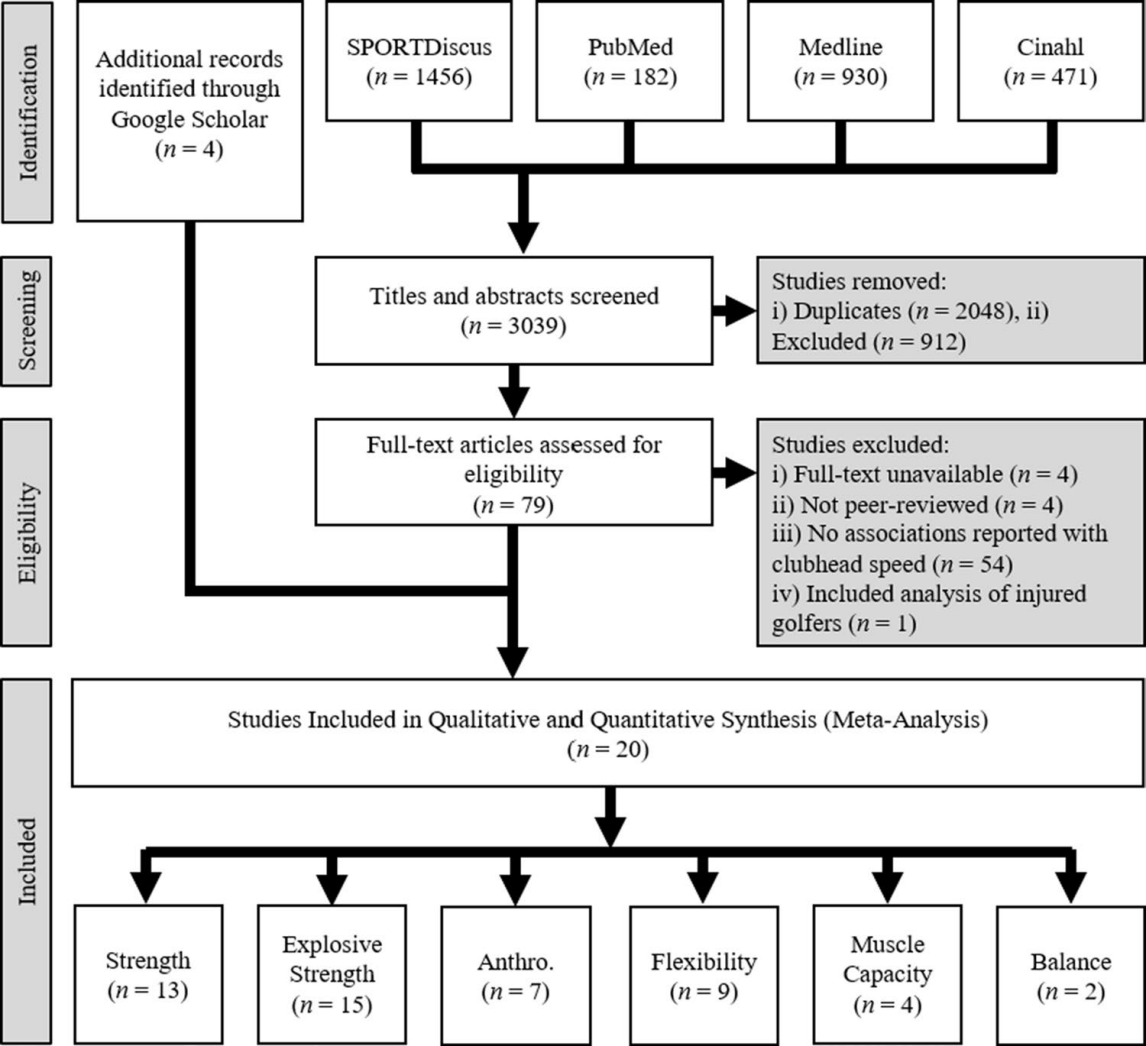
Flow diagram showing the identification and selection of studies. Included studies often reported more than one physical characteristic, which is why the bottom row of numbers (*n* = 50) is greater than the total number of studies (*n* = 20). *Anthro.* Anthropometry

The present study was undertaken in line with the recommendations of the Preferred Reporting Items for Systematic Review and Meta-Analysis (PRISMA) guidelines [17]. Four databases (Medline, SPORTDiscus, CINAHL and PubMed) were electronically searched to gather relevant literature, and Fig. 1 provides a schematic overview of the search methodology, conducted in January 2023, with no restrictions on the publication date. A search strategy was used within Boolean operators in order to identify specific articles relevant to the research question, with a summary provided in Table 1.

**Table 1:** An overview of the specific words and phrases used for search terms

Operator	Search term order	Search term(s)
	#1	Golf
AND	#2	Physical
AND	#3	Strength
AND	#4	Power
AND	#5	Fitness
AND	#6	Flexibility
AND	#7	Speed
AND	#8	Velocity
AND	#9	Relationship
AND	#10	Association

### Inclusion/Exclusion Criteria

Inclusion criteria required studies to have (1) determined the association between physical characteristics assessed in at least one physical test and CHS; (2) included golfers of any skill level, but they needed to be free from injury; and (3) been peer-reviewed and published in the English language. Studies were excluded if they were (1) reviews or conference articles, (2) intervention studies and (3) if they did not contain original data. After completing all relevant searches, an additional search was completed in Google Scholar for any articles that may have been relevant or not fully available in the aforementioned databases. Finally, reference lists of included studies, alongside forward citations, were also searched for relevant articles.

### Screening Strategy

The articles extracted during the search strategy were then screened through a three-stage process: (1) duplicates of articles from previous search terms and databases were removed, (2) articles that were considered potentially appropriate were passed through for a full review and (3) articles were reviewed in full in line with the inclusion and exclusion criteria by two independent reviewers (AB and CB). If any disagreement occurred between reviewers one and two, a third reviewer (AE) was consulted to resolve this issue.

### Grading Article Quality

To assess the quality of the study’s methodology, a modified version of the Downs and Black Quality Index tool [18] was used, in accordance with previous studies [19, 20]. From the original version, questions were removed for this current review if they were deemed irrelevant (not applicable to the research question). Specifically, questions associated with patient treatment, training interventions and group randomisation processes were removed. Following this, ten questions in the checklist were deemed relevant (Table 2). Each question was given a score of 1 ('+') or 0 ('−'), or ('?') if it was unable to be determined, by two reviewers (AB and CB), with a total score out of 10 possible for each study. If a consensus on the score could not be reached, a third reviewer (AE) was consulted to resolve this issue.

**Table 2:** Questions chosen from the Downs and Black [18] checklist used to evaluate the methodological quality of included studies

Question number	Question
Reporting	
1	Is the hypothesis/aim/objective of the study clear?
2	Are the main outcomes to be measured clearly described in the introduction or methods section? *Information outlined in introduction/methodology for both physical characteristics and golf performance measure used for associative analysis pertaining to assessment(s) used, any calculations used and units of measurement
3	Are the characteristics of the subjects included in the study clearly described? *Skill level of golfers and additional characteristics included
4	Are the main findings of the study clearly described?
5	Does the study provide estimates of the random variability in the data for the main outcomes? *One of the following included for both physical characteristics and golf performance measures: (a) mean ± SD, (b) standard error, (c) confidence intervals and (d) interquartile range
6	Have actual probability values been reported (e.g. 0.035 rather than < 0.05) for the main outcomes except where the probability value is less than 0.001? *Exact correlation (*r*) and significance (*p*) values provided, specific to the associative analysis
External validity	
7	Were the subjects to participate in the study representative of the entire population from which they were recruited? * Proportion of subjects asked to participate, relative to the sample population, explicitly stated. Unless evident, then answer ‘unable to determine’
Internal validity	
8	If any of the results of the study were based on ‘data dredging,’ was this made clear? *Were any additional data analysis reported in the results not highlighted during the methodology?
9	Were statistical tests used to assess the main outcomes appropriate?
10	Were the main outcome measures accurate (valid and reliable)?

### Statistical Analysis

Initially, key data were directly extracted from studies that met the inclusion criteria and transferred into Microsoft Excel. For all studies, key information extracted included the following: (1) sample population, (2) physical assessment(s), (3) CHS data and (4) correlation value(s) between physical assessment(s) and CHS. To provide summary effects for each of the physical characteristics, a random effects model was used, which accounts for the magnitude of the standard error associated with each of the included studies (due to different methodologies, skill level of samples, etc.). To achieve this, *z*-transformed *r* values (i.e. *z*_*r*_ values) were computed to enable effect size pooling within the meta-analysis [21]. Where studies reported multiple eligible effect sizes for any one physical attribute (of which inclusion would violate the assumption of independence within the meta-analysis model), these were pooled by first transforming to Fisher’s *Z* (*z*_*r*_) values, aggregating an average, and back transforming to a Pearson’s *r* value for input within the meta-analysis. In instances where multiple clubs were reported within a single study, we opted to use driver CHS as the metric to carry forwards, for consistency. Lastly, where directional differences were found in the pooled data of a single study (e.g. where included metrics comprised both values where a larger value is considered more favourable and values where a smaller value is considered more favourable), the negatively aligned data were inverted by multiplying by − 1, to directionally align all metrics prior to pooling. The meta-analysis was performed using the ‘metafor’ package (version 4.4-0) in R (version 4.2.2; R core team), and effect size values were interpreted in line with suggestions by Cohen [22], whereby < 0.2 = trivial, 0.2–0.49 = small, 0.5–0.79 = moderate and ≥ 0.8 = large.

### Stability and Validity of Changes in Effect Sizes

To assess for the presence and degree of heterogeneity in the data, both *Q* statistics and *I*^2^ were used. Statistical significance for *Q* was acknowledged at an alpha level of < 0.10, and *I*^2^ was interpreted as per the work of Higgins and Thompson [23], with an *I*^2^ threshold of 0–25% = trivial, 25–50% = low, 50–75% = moderate and 75–100% = high. Small study bias (including publication bias) was assessed firstly by the visualisation of funnel plots, and accompanied by the Egger’s regression test to quantify any asymmetries in the spread of data, and thus risk of small study bias. The occurrence of small study bias was considered present where *p* < 0.05, and in these circumstances, the trim and fill method was used to artificially impute potentially missing studies due to asymmetry in the funnel plot, and provide an adjusted pooled effect size to account for this [24].

## Results

### Literature Search Results

The search strategy produced a total of 3039 studies, of which 2048 were removed due to being duplicates. In total, 16 studies met our inclusion criteria (Fig. 1) with four additional studies included following reference list checks and forward citation tracking, resulting in a total of 20 included studies. Once full texts were assessed for eligibility, the most common reasons for exclusion were (1) no relationship determined between physical characteristics and CHS (or the *r* value was not reported), (2) articles were not peer-reviewed, (3) full texts were unavailable and (4) the sample included golfers who were recovering from injury.

### Methodological Quality and Risk of Bias Assessment

Study methodological quality is presented in Table 3. There was no evidence of internal validity bias. We were unable to confirm external validity in all the studies analysed, with all failing to report the proportion of individuals recruited relative to the sample population. Scores ranged from 7 to 9 out of 10 for study methodological quality and risk of bias, with no studies excluded due to their chosen methodological approach.

**Table 3:** Results of study methodological quality for all included studies, using the checklist from Downs and Black [18]

Study	Checklist item number										Total score out of 10
	Reporting						External validity	Internal validity			
	1	2	3	4	5	6	7	8	9	10	
Brown et al. [15]	+	+	+	+	+	−	?	+	+	+	8
Coughlan et al. [27]	+	+	+	+	+	−	?	+	+	+	8
Donahue et al. [29]	+	+	+	+	+	−	?	+	+	+	8
Gordon et al. [33]	+	+	+	+	+	−	?	+	+	+	8
Hellström [14]	+	+	−	+	+	−	?	+	+	+	7
Keogh et al. [34]	+	+	+	+	+	+	?	+	+	+	9
Leary et al. [35]	+	+	+	+	+	+	?	+	+	+	9
Lewis et al. [10]	+	+	+	+	+	−	?	+	+	+	8
Loock et al. [36]	+	+	+	+	+	+	?	+	+	+	9
Marshall and Llewellyn [30]	+	+	+	+	+	−	?	+	+	+	8
Oranchuk et al. [31]	+	+	+	+	+	+	?	+	+	+	9
Parchmann and McBride [32]	+	+	+	+	+	+	?	+	+	+	9
Read et al. [37]	+	+	+	+	+	−	?	+	+	+	8
Sheehan et al. [38]	+	+	+	+	+	−	?	+	+	+	8
Sorbie et al. [39]	+	+	+	+	+	+	?	+	+	+	9
Suhara et al. [40]	+	+	+	+	+	+	?	+	+	+	9
Sanders et al. [28] Wells et al. [25] Wells et al. [26] Wells et al. [41]	+ + + +	+ + + +	+ + + +	+ + + +	+ + + +	+ + + +	? ? ? ?	+ + + +	+ + + +	+ + + +	9 9 9 9

+ yes; − no, ?  unable to determine

### Study Characteristics

Information on each study included in the final analysis is presented in Table 4. Sample populations included the following: (1) Category 1 golfers (*n* = 93) (15,25,26); (2) elite youth golfers (*n* = 82) [27, 28]; (3) National Collegiate Athletics Association (NCAA) Division I and II golfers (*n* = 61) [29–32]; (4) single- and double-digit handicap golfers (*n* = 282) [14, 33–35, 37–40]; (5) European Tour players (*n* = 31) [41]; and (6) PGA tour players (*n* = 20) [10]. From the final 20 studies, associations between CHS and physical characteristics included strength (*n* = 13) [14, 15, 26, 28, 29, 31–36, 38, 40], explosive strength (*n* = 14) [10, 14, 25–27, 31–33, 35, 37–41], anthropometrics [14, 27, 29, 34, 37, 38], flexibility [15, 30, 32, 33, 36, 38], muscle capacity (endurance) [14, 27, 34, 36] and balance [30, 36].

**Table 4:** Overview of study methodologies and raw data for physical characteristics

Study	Sample	Physical assessments	Physical assessment data
Brown et al. [15]	16 Category 1 golfers (handicap: ≤ 5) Females (*n* = 16)	Grip strength (kg⋅f-1), standing flexibility, clockwise and counter-clockwise (m), sitting flexibility, clockwise and counter-clockwise (m)	Grip strength (left: 32.94 ± 5.26; right: 35.25 ± 5.93 kg⋅f-1); standing flexibility (clockwise: 0.39 ± 0.2; anti-clockwise: 0.42 ± 0.2 m); seated flexibility (clockwise: 0.62 ± 0.15; anti-clockwise: 0.58 ± 0.13 m)
Coughlan et al. [27]	69 England national, regional or county level youth golfers (Handicap: 1.8 ± 2.4) Males (*n* = 36) Females (*n* = 33)	Mass (kg), height (cm), CMJ (cm), CMJ power (W), standing long jump (m), seated med-ball throw right and left (m), rotational med-ball throw right and left (m), push-up (reps) and modified pull-up (reps)	Mass (68.6 ± 8.5 kg); height (176.7 ± 7.1 cm); CMJ height (32.5 ± 8.0 cm); CMJ power (3027.8 ± 511.8 W); standing long jump (2.1 ± 0.2 m); seated med-ball throw (left: 3.9 ± 0.8; right: 4.5 ± 1.0 m); rotational med-ball throw (left: 8.2 ± 1.8; right: 8.3 ± 1.9 m); push-ups (10.0 ± 4.4 reps); and modified pull-up (9.0 ± 4.2 reps)
Donahue et al. [29]	14 Division I collegiate golfers (Sex = not reported)	Height (cm), body mass (kg), vertical jump (cm), grip strength (kg), rotational medicine ball toss (m/s) and sit and reach (cm)	Height (179.30 ± 7.20 cm); body mass (76.09 ± 11.61 kg); vertical jump height (54.43 ± 7.42 cm); grip strength (104.64 ± 15.93 kg); rotational medicine ball toss (9.16 ± 0.72 m/s); and sit and reach (26.36 ± 6.46 cm)
Gordon et al. [33]	15 golfers (Handicap: 4.9 ± 2.9) Males (*n* = 15)	Pec-dec resistance machine (8RM), 3-kg rotational hip toss (m) and rotational trunk flexibility (°)	Pec-dec resistance machine (44.5 ± 10.9 kg); 3-kg rotational hip toss (8.0 ± 0.9 m) and rotational trunk flexibility (71.8 ± 11.8°)
Hellström et al. [14]	33 elite golfers (Estimated handicap: 5 to 0) Males (*n* = 33)	Body mass (kg), bar dips (reps), pull-ups (reps), vertical sit-ups (reps), bar dips (kg), pull-ups (kg), vertical sit-ups (kg) grip left and right (kg), squat (kg), squat jump height (cm), CMJ height (cm), CMJ with arm swing (cm), squat jump peak power (W), CMJ peak power (W), CMJ with arm swing peak power (W), sprint time 10 and 20 m (s) and sprint 10 and 20 m mean power (W)	Body mass (75.5 ± 7.3 kg); bar dips (13.8 ± 6.2 reps); pull-ups (7.1 ± 4.1 reps); vertical sit-ups (15.9 ± 5.6 reps), bar dips (1041 ± 460 kg); pull-ups (528 ± 300 kg); vertical sit-ups (597 ± 208 kg); grip relative (left: 0.69 ± 0.10; right: 0.73 ± 0.08); squat relative (1.49 ± 0.28); grip (left: 52.2 ± 8.7; right: 55.0 ± 7.7 kg); squat (112 ± 25 kg); squat jump height (30.3 ± 4.9 cm); CMJ height (32.9 ± 5.2 cm); CMJ average height (39.2 ± 5.9 cm); squat jump peak power (3202 ± 494 W); CMJ peak power (3357 ± 519 W); CMJ average peak power (3743 ± 538 W); sprint 10 m time (1.73 ± 0.07 s); sprint 20 m time (3.10 ± 0.12 s); sprint 10 m mean power (1458 ± 217 W); and sprint 20 m mean power (1023 ± 147 W)
Keogh et al. [34]	20 low- and high-handicap golfers (Low handicap: 0.3 ± 0.5) (High handicap: 20.3 ± 2.4) Males (*n* = 20)	Height (m), body mass (kg), body mass index (kg⋅${{\text{m}}}^{-2}$), sum of 4 skinfolds (mm), body fat (%), fat mass (kg), fat-free mass (kg), acromiale-radiale length (cm), radiale-stylion length, acromiale-stylion length (cm), biacromial width (cm), chest girth (cm), upper arm girth (cm), follow through trunk rotation (°), backswing trunk rotation, back hand wrist abduction and adduction (°), front leg hip internal and external rotation (°), back leg hip internal and external rotation, golf swing cable woodchop (kg), bench press (kg), hack squat (kg) and isometric prone hold (s)	**Low handicap golfers:** Height (1.80 ± 0.07 cm); body mass (76.8 ± 8.8 kg), body mass index (26.8 ± 2.9 kg⋅${{\text{m}}}^{-2}$); sum of 4 skinfolds (31.3 ± 10.0 mm); body fat (10.0 ± 2.9%); fat mass (7.8 ± 2.9 kg); fat-free mass (69.0 ± 6.8 kg); acromiale-radiale length (34.1 ± 1.5 cm); radiale-stylion length (26.8 ± 1.2 cm); acromiale-stylion length (60.9 ± 2.5 cm); biacromial width (42.1 ± 1.5 cm); chest girth (96.2 ± 5.1 cm); upper arm girth (31.8 ± 2.2 cm); follow through trunk rotation (68 ± 12°); backswing trunk rotation (70 ± 12°); back hand wrist adduction (40 ± 4°); back hand wrist abduction (22 ± 7°); front leg hip internal rotation (32 ± 7°); back leg hip internal rotation (26 ± 7°); front leg hip external rotation (23 ± 7°); back leg hip external rotation (32 ± 6°); golf swing cable woodchop (68.9 ± 9.2 kg); bench press (76.0 ± 17.5 kg); hack squat (95.6 ± 25.8 kg); and isometric prone hold (173 ± 88 s) **High handicap golfers:** Height (1.77 ± 0.07 cm); body mass (73.5 ± 11.8 kg), body mass index (26.8 ± 4.7 kg⋅${{\text{m}}}^{-2}$); sum of 4 skinfolds (39.7 ± 18.3 mm); body fat (12.5 ± 5.5%); fat mass (9.7 ± 5.7 kg); fat-free mass (63.8 ± 7.3 kg); acromiale-radiale length (32.5 ± 1.7 cm); radiale-stylion length (25.9 ± 1.2 cm); acromiale-stylion length (58.4 ± 2.6 cm); biacromial width (42.1 ± 2.4 cm); chest girth (93.7 ± 6.7 cm); upper arm girth (30.6 ± 2.7 cm); follow through trunk rotation (58 ± 16°); backswing trunk rotation (60 ± 12°); back hand wrist adduction (39 ± 8°); back hand wrist abduction (21 ± 8°); front leg hip internal rotation (34 ± 5°); back leg hip internal rotation (34 ± 8°); front leg hip external rotation (28 ± 7°); back leg hip external rotation (31 ± 8°); golf swing cable woodchop (53.7 ± 7.0 kg); bench press (58.6 ± 15.2 kg); hack squat (82.7 ± 12.8 kg); and isometric prone hold (105 ± 64 s)
Leary et al. [35]	12 recreational golfers (Handicap: 14.5 ± 7.3) Males (*n* = 12)	IMTP: Force (N), allometrically scaled isometric force (N⋅k ${{\text{g}}}^{-0.67}$), rate of force development (N⋅${{\text{s}}}^{-1}$) CMJ: peak force (N), allometrically scaled peak force (N⋅k ${{\text{g}}}^{-0.67}$), relative peak force (N⋅${{\text{kg}}}^{-1}$), peak rate of force development (N⋅${{\text{s}}}^{-1}$), peak velocity (m⋅${{\text{s}}}^{-1})$, peak power (W), allometrically scaled peak power (W⋅k ${{\text{g}}}^{-0.67}$), relative peak power (W⋅k ${{\text{g}}}^{-1}$), average power (W), peak rate of power development (W⋅${{\text{s}}}^{-1}$), vertical displacement (m) Static vertical jump: peak force (N), allometrically scaled peak force (N⋅k ${{\text{g}}}^{-0.67})$, relative peak force (N⋅k ${{\text{g}}}^{-1})$, peak rate of force development (N⋅${{\text{s}}}^{-1}$), peak velocity (m⋅${{\text{s}}}^{-1})$, peak power (W), allometrically scaled peak power (W⋅k ${{\text{g}}}^{-0.67}$), relative peak power (W⋅k ${{\text{g}}}^{-1}$), average power (W), peak rate of power development (W⋅${{\text{s}}}^{-1}$), vertical displacement (m) Eccentric utilisation ratio of power Eccentric utilisation ratio of displacement	IMTP: Isometric force (N) at force at 30 ms (47.9 ± 24.5); 50 ms (183.3 ± 87.9); 90 ms (535.8 ± 254.7); 100 ms (594.9 ± 261.0); 150 ms (849.7 ± 314.1); 200 ms (1188.7 ± 405.4); 250 ms (1401.4 ± 350.4); and peak (2137.8 ± 323.4) Allometrically scaled isometric force (N⋅k ${{\text{g}}}^{-0.67})$ at 30 ms (2.5 ± 1.3); 50 ms (9.9 ± 5.1); 90 ms (29.2 ± 15.7); 100 ms (32.4 ± 15.9); 150 ms (45.3 ± 14.0); 200 ms (63.6 ± 19.3); 250 ms (75.8 ± 18.8); and peak (116.8 ± 25.7) Rate of force development (N⋅${{\text{s}}}^{-1}$) at 0–30 ms (2831.1 ± 1578.5); 0–50 ms (4319.6 ± 2356.4); 0–90 ms (6364.6 ± 3136.5); 0–100 ms (6319.6 ± 2861.0); 0–150 (5911.7 ± 2032.8); 0–200 ms (6128.6 ± 2002.7); 0–250 ms (5753.8 ± 1460.9); and peak (12,393.1 ± 5323.1) CMJ: Peak force (1685.5 ± 196.9 N); allometrically scaled peak force (93.6 ± 4.1 N⋅k ${{\text{g}}}^{-0.67}$), relative peak force (22.1 ± 0.9 N⋅${{\text{kg}}}^{-1}$); peak rate of force development 7624.4 ± 1920.5 N⋅${{\text{s}}}^{-1}$); peak velocity (3.1 ± 0.3 m⋅${{\text{s}}}^{-1});$ peak power (4693.4 ± 805.3 W); allometrically scaled peak power (260.3 ± 34.2 W⋅k ${{\text{g}}}^{-0.67}$); relative peak power (61.5 ± 7.7 W⋅k ${{\text{g}}}^{-1});$ average power (1681.5 ± 344.5 W); peak rate of power development (21,550.8 ± 5049.4 (W⋅${{\text{s}}}^{-1})$; and vertical displacement (0.38 ± 0.07 m) Static vertical jump: Peak force (14,780.0 ± 205.0 N); allometrically scaled peak force (82.2 ± 8.1 N⋅k ${{\text{g}}}^{-0.67});$ relative peak force (19.4 ± 1.9 N⋅k ${{\text{g}}}^{-1});$ peak rate of force development (5184.5 ± 2584.4 (N⋅${{\text{s}}}^{-1});$ peak velocity (2.9 ± 0.3 m⋅${{\text{s}}}^{-1});$ peak power (4093.6 ± 647.1 W); allometrically scaled peak power (227.8 ± 30.2 W⋅k ${{\text{g}}}^{-0.67}$); relative peak power (53.8 ± 7.1 W⋅k ${{\text{g}}}^{-1})$; average power (1999.7 ± 404.1 W); peak rate of power displacement (12,971.7 ± 5272.1 W⋅${{\text{s}}}^{-1}$); vertical displacement (0.32 ± 0.06 m) Eccentric utilisation ratio of power (1.15 ± 0.14) Eccentric utilisation ratio of displacement (1.19 ± 0.11)
Lewis et al. [10]	20 members of the PGA golfers Males (*n* = 20)	Squat jump (cm), rotational medicine ball throw (cm), seated medicine ball throw (cm)	Squat jump height (33.4 ± 6.48 cm); rotational medicine ball throw (762 ± 107.06 cm); and seated medicine ball throw (580.5 ± 49.36)
Loock et al. [36]	101 ‘experienced’ golfers (Handicap: not reported) Males (*n* = 101)	Step test (heart rate), sit-ups (per minute), push-ups (per minute), lower back strength (kg), wall squats (per minute) and balance (Biodex system score)	Step test (126.50 ± 19.97 HR); sit-ups (37.59 ± 9.72 reps); push-ups (25.99 ± 11.44 reps); lower back strength (143.90 ± 39.08 kg); wall squats (34.56 ± 9.76 reps); and balance (3.08 ± 2.25 Biodex System Score)
Marshall and Llewellyn [30]	10 Nebraska Wesleyan University golfers (Handicap: 2–22) Males (*n* = 5) Females (*n* = 5)	Sit-and-reach max (cm) and Balance Error Scoring System test (errors)	Male: Sit-and-reach max (32.0 ± 7.4 cm) and Balance Error Scoring System test (12.8 ± 6 errors) Female: Sit-and-reach max (34.6 ± 4.0 cm) and Balance Error Scoring System test (15.4 ± 4 errors)
Oranchuk et al. [31]	12 NCAA Division II collegiate golfers Males (*n* = 6) Females (*n* = 6)	1RM back squat (kg), 1RM deadlift (kg), 1RM clean (kg), average CMJ height (cm) and peak CMJ height (cm)	1RM back squat (115.3 ± 42.8 kg); 1RM deadlift (131.2 ± 44.3 kg); 1RM clean (67.5 ± 19.4 kg); average CMJ height (54.0 ± 9.0 cm); and peak CMJ height (55.0 ± 9.5 cm)
Parchmann and McBride [32]	25 NCAA Division I golfers Males (*n* = 15) Females (*n* = 10)	7 functional movement screen tests (0 to 3), 1RM squat, vertical jump height (cm), sprint 10 m and 20 m (s) and agility T test (s)	Not reported
Read et al. [37]	48 golfers (Handicap: 5.8 ± 2.2) Males (*n* = 48)	CMJ height (cm), CMJ peak power (W), squat jump height (cm), squat jump peak power (W), right leg CMJ height (cm), left leg CMJ height (cm), medicine ball seated throw (m), medicine ball rotational throw (m), standing height (m), weight (kg) and arm length (cm)	CMJ height (28.4 ± 4.6 cm); CMJ peak power (3094 ± 609 W); squat jump height (27.1 ± 4.2 cm); squat jump peak power (3015 ± 630 W); right leg CMJ height (14.4 ± 3.1 cm); left leg CMJ height (14.4 ± 2.8 cm); medicine ball seated throw (4.7 ± 0.8 m); medicine ball rotational throw (9.6 ± 1.7 m); standing height (1.8 ± 0.1 m); weight (75.6 ± 13.5 kg); and arm length (58.4 ± 3.4 cm)
Sheehan et al. [38]	22 golfers (Handicap: 9.89 ± 8.36) Males (*n* = 22)	Height (cm), weight (kg), body mass index and age (years) Power and strength: CMJ height (cm), CMJ peak power (W⋅k ${{\text{g}}}^{-1}$), squat jump height (cm), squat jump peak power (W⋅k ${{\text{g}}}^{-1}$), eccentric utilisation ratio (%), countermovement push-up peak force (N), countermovement push-up peak rate of force development (N⋅${{\text{s}}}^{-1}$), concentric-only push-up peak force (N), concentric-only push-up peak rate of force development (N⋅s^−1^), concentric-only push-up power (W⋅kg^−1^), medicine ball throw velocity (km⋅h^−1^), lead grip strength (kg), trail grip strength (kg), isometric squat peak force (N), isometric squat peak rate of force development (N⋅s^−1^), isometric push-up peak force (N), isometric push-up peak rate of force development (N⋅s^−1^) Stiffness (N⋅m^−1^): lead and trail bicep femoris stiffness, lead and trail vastus lateralis, lead and trail external oblique, lead and trail pectoralis major, lead and trail latissimus dorsi, trail flexor carpi ulnaris, lead vertical and trail vertical Flexibility: sit and reach lead and trail leg (cm), torso rotation lead and trail side (°), hip internal rotation lead and trail side (°), hip external rotation lead and trail side (°)	Height (180 ± 5.56 cm); weight (81.6 ± 7.72 kg); and body mass index (25.1 ± 2.16) Power and strength: CMJ height (34.6 ± 3.87 cm); CMJ peak power (50.9 ± 4.68 W⋅k ${{\text{g}}}^{-1}$); squat jump height (31.2 ± 4.85 cm); squat jump peak power (49.7 ± 4.82 W⋅k ${{\text{g}}}^{-1}$); eccentric utilisation ratio (11.9 ± 13.3%); countermovement push-up peak force (1014 ± 160 N); countermovement push-up rate of force development (5003 ± 147 N.${{\text{s}}}^{-1}$); concentric-only push-up peak force (870 ± 124 N); concentric-only push-up peak rate of force development (4437 ± 1654 N⋅s^−1^); concentric-only push-up power (11.1 ± 2.96 W⋅kg^−1^); medicine ball throw velocity (23.3 ± 2.78 km⋅h^−1^); lead grip strength (37.6 ± 6.56 kg); trail grip strength (40.4 ± 6.66 kg); isometric squat peak force (1711 ± 323 N); isometric peak rate of force development (5932 ± 2732 N⋅s^−1^); isometric push-up peak force (614 ± 262 N); and isometric push-up peak rate of force development (2802 ± 1471 N⋅s^−1^) Stiffness (N⋅m^−1^): Lead bicep femoris (350 ± 45.0); trail bicep femoris (350 ± 48.9); lead vastus lateralis (405 ± 83.4); trail vastus lateralis (402 ± 60.9); trail external oblique (194 ± 22.24); lead pectoralis major (207 ± 17.5); trail pectoralis major (210 ± 23.1); lead latissimus dorsi (202 ± 28.8); trail latissimus dorsi (206 ± 35.5); trail flexor carpi ulnaris (350 ± 70.5); lead vertical stiffness (11,704 ± 2137); and trail vertical stiffness (11,275 ± 1307) Flexibility: Lead sit and reach (22.0 ± 9.9 cm); trail sit and reach (21.9 ± 9.7 cm); lead torso rotation (33.5 ± 10.9°); trail torso rotation (27.6 ± 10.2°); lead external hip rotation (35.4 ± 7.4°); lead internal hip rotation (23.7 ± 8.1°); trail external hip rotation (34.7 ± 9.2°); and trail internal hip rotation (19.3 ± 5.0°)
Sorbie et al. [39]	13 skilled golfers (Handicap: 6.1 ± 4.9) (Sex = not reported)	Ballistic bench press: Peak power (W), mean power (W), relative peak power (W·kg^−1^) and relative mean power (W·kg^−1^) Wingate test: Peak power (W), mean power (W), relative peak power (W·kg^−1^) and relative mean power (W·kg^−1^)	Ballistic bench press: Peak power (631.50 ± 153.27 W); mean power (323.58 ± 85.98 W); relative peak power (8.14 ± 2.33 W·kg^−1^); and relative mean power (4.17 ± 1.22 W·kg^−1^) Wingate test: Peak power (396.49 ± 89.23 W); mean power (293.18 ± 61.53 W); relative peak power (5.06 ± 1.08 W·kg^−1^); and relative mean power (3.74 ± 0.70 W·kg^−1^)
Suhara et al. [40]	28 elite amateur golfers (Handicap: males = 4.4 ± 6 0.9; females = 6.2 ± 6 0.7) Males (*n* = 14) Females (*n* = 14)	Biodex (60°·s^−1^, N·m $$;$$ 180°·s^−1^, N·m$$)$$: Hip extension right and left, hip flexion right and left, torso rotation right and left CMJ height (cm) CMJ power (W) BOMB throw (m)	Males: Biodex (60°·s^−1^, N·m): hip extension (right: 238.9 ± 49.2; left: 222.8 ± 32.9); hip flexion (right: 152.4 ± 18.3; left: 154.4 ± 19.4); and torso rotation (right: 140.3 ± 22.8; left: 142.4 ± 28.1) Biodex (180°·s^−1^, N·m): hip extension (right: 212.2 ± 30.2; left: 198.6 ± 27.6); hip flexion (right: 126.1 ± 16.6; left: 131.5 ± 12.5); and torso rotation (right: 126.5 ± 21.2; left: 129.4 ± 23.0) CMJ height (46.6 ± 6.0 cm) CMJ power (4187 ± 371.2 W) BOMB throw (10.8 ± 1.2 m) Females: Biodex (60°·s^−1^, N·m): hip extension (right: 175.1 ± 23.5; left: 159.9 ± 18.8); hip flexion (right: 103.6 ± 12.6; left: 105.3 ± 9.5); and torso rotation (right: 87.7 ± 13.9; left: 88.7 ± 9.5) Biodex (180°·s^−1^, N·m): hip extension (right: 148.1 ± 19.1; left: 140.7 ± 17.9); hip flexion (right: 86.2 ± 15.1; left: 90.0 ± 13.5); and torso rotation (right: 79.6 ± 12.3; left: 82.1 ± 11.0) CMJ height (36.7 ± 4.5 cm) CMJ power (2673.3 ± 266.4 W) BOMB throw (8.4 ± 1.2 m)
Sanders et al. [28]	13 high-level youth golfers (Handicap: 2.7 ± 3.0) Males (*n* = 7) Females (*n* = 6)	IMTP: Peak force (N), force at 50 ms (N), force at 100 ms (N), force at 150 ms (N), force at 200 ms (N), force at 200 ms (N), force at 250 ms (N), peak rate of force development (N⋅${{\text{s}}}^{-1}$), rate of force development 0–50 ms (N⋅${{\text{s}}}^{-1}$), rate of force development 0–100 ms (N⋅${{\text{s}}}^{-1}$), rate of force development 0–150 (N⋅${{\text{s}}}^{-1}$), rate of force development 0–200 (N⋅${{\text{s}}}^{-1}$), rate of force development 0–250 (N⋅${{\text{s}}}^{-1}$)	IMTP: Force (N): peak force (1399.2); force at 50 ms (251.3); force at 100 ms (452.1); force at 150 ms (603.3); force at 200 ms (862.2); and force at 250 ms (933.2) Rate of force development (N⋅${{\text{s}}}^{-1}$): peak (7477.3); 0–50 ms (4014.6); 0–100 ms (3809.5); 0–150 ms (3776.8); 0–200 ms (4172.0); and 250 ms (3584.9)
Wells et al. [25]	50 Category 1 golfers (Handicap: 2.9 ± 1.9) Males (*n* = 50)	CMJ: Net impulse (N⋅s), positive impulse (N⋅s), average power (W), peak power (W), peak force (N), force at zero velocity (N) and jump height (m)	Net impulse (180.92 ± 26.00 N⋅s); positive impulse (282.70 ± 42.98 N⋅s); average power (1771.94 ± 295.08 W); peak power (3435.32 ± 553.51 W); peak force (876.21 ± 158.67 N); force at zero velocity (821.86 ± 188.41 N); and jump height (0.30 ± 0.06 m)
Wells et al. [26]	27 Category 1 golfers (Handicap: − 2.7 ± 1.9) Males (*n* = 27)	IMTP: Peak force (N), rate of force development 0–50 (N/s), rate of force development 0–100 (N/s), rate of force development 0–150 (N/s) and rate of force development 0–200 (N/s) CMJ positive impulse (N⋅s) Drop jump positive impulse (N⋅s) Squat jump positive impulse (N⋅s)	IMTP: Peak force (1604.57 ± 391.47 N); rate of force development 0–50 (3604.3 ± 1986.75 N/s); rate of force development 0–100 (4864.13 ± 2331.42 N/s); rate of force development 0–150 (5018.06 ± 1787.84 N/s); rate of force development 0–200 (4863.60 ± 1459.12 N/s) CMJ positive impulse (286.22 ± 42.19 N⋅s) Drop jump positive impulse (423.43 ± 58.3 N⋅s) Squat jump positive impulse (185.05 ± 29.04 N⋅s)
Wells et al. [41]	31 European Challenge Tour golfers Male (*n* = 31)	IMTP: Peak force (N), rate of force development 0–50 (N/s), rate of force development 0–100 (N/s), rate of force development 0–150 (N/s), rate of force development 0–200 (N/s) CMJ peak impulse (N⋅s)	IMTP: Peak force (2093.31 ± 365.97 N); rate of force development 0–50 (7833.04 ± 5530.74 N/s); rate of force development 0–100 (6109.92 ± 3073.52 N/s); rate of force development 0–150 (5680.65 ± 2466.21 N/s); rate of force development 0–200 (6064.91 ± 2123.18 N/s) CMJ peak impulse (279.81 ± 46.85 N·s)

*BOMB* backward overhead medicine ball, *cm* centimetres, *CMJ* countermovement jump, *HR* heart rate, *IMTP* isometric mid-thigh pull, *kg* kilograms, *m* metres, *m/s* metres per second, *N* Newtons, *NCAA* National Collegiate Athletics Association, *N.kg*^*−1*^ Newtons per kilogram, *N/s* Newtons per second, *N·m* Newton metres, *N·s* Newton seconds, *º* degrees, *pec dec* resistance machine primarily involving use of the pectoralis major muscles, *PGA* Professional Golfers’ Association, *reps* repetitions, *RM* repetition maximum, *s* seconds, *W* watts

### Meta-Analysis

A breakdown of each physical characteristic and its summary statistics are provided, with Figs. 2, 3, 4, 5, 6, 7, 8, 9, 10 and 11 visualising the data via forest plots, which represent the summary effect estimate relative to the inputed samples.

**Fig. 2:**
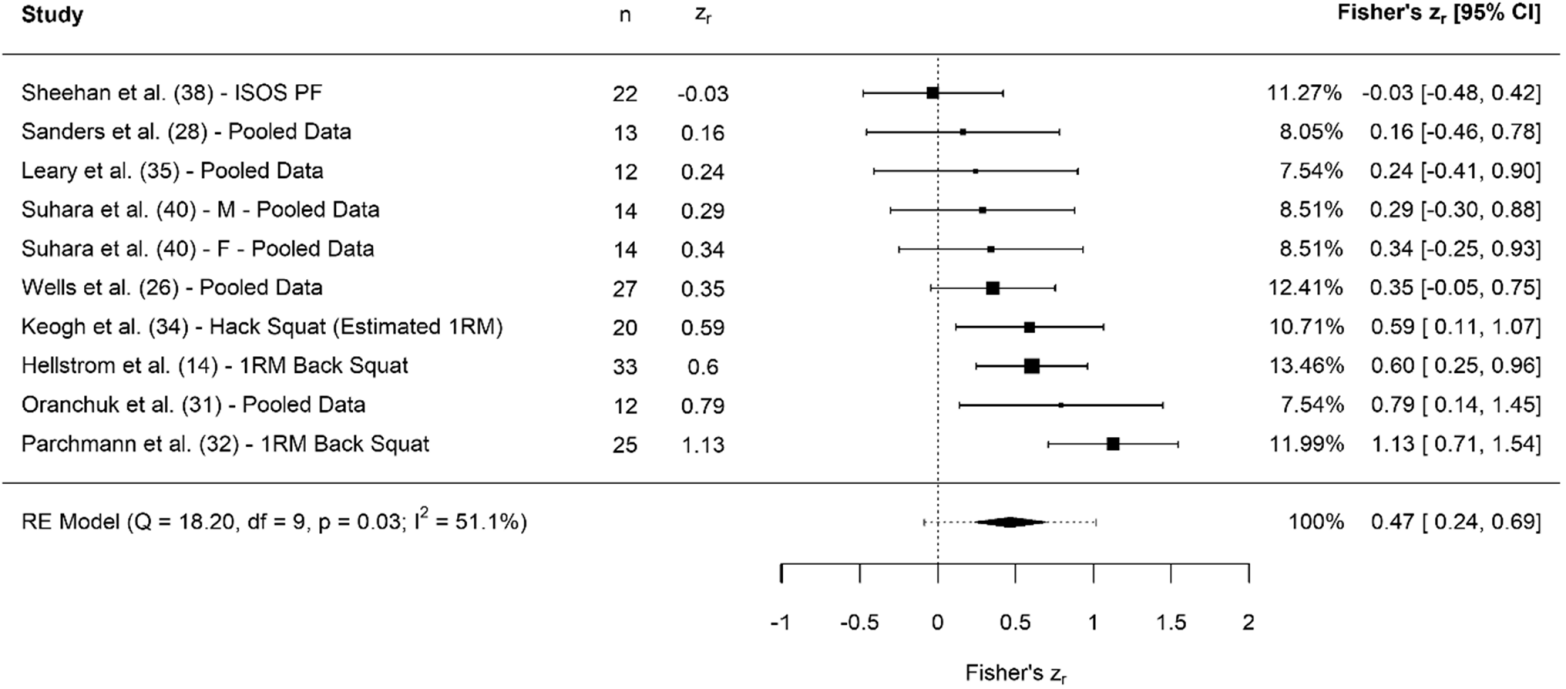
Forest plot showing the association (Fisher’s *z*_*r*_) between lower body strength and clubhead speed. *n* = sample size. *CI* confidence interval, *F* females, *ISOS* isometric squat, *M* males, *PF* peak force, *RE* random effects, *RM* repetition maximum

**Fig. 3:**
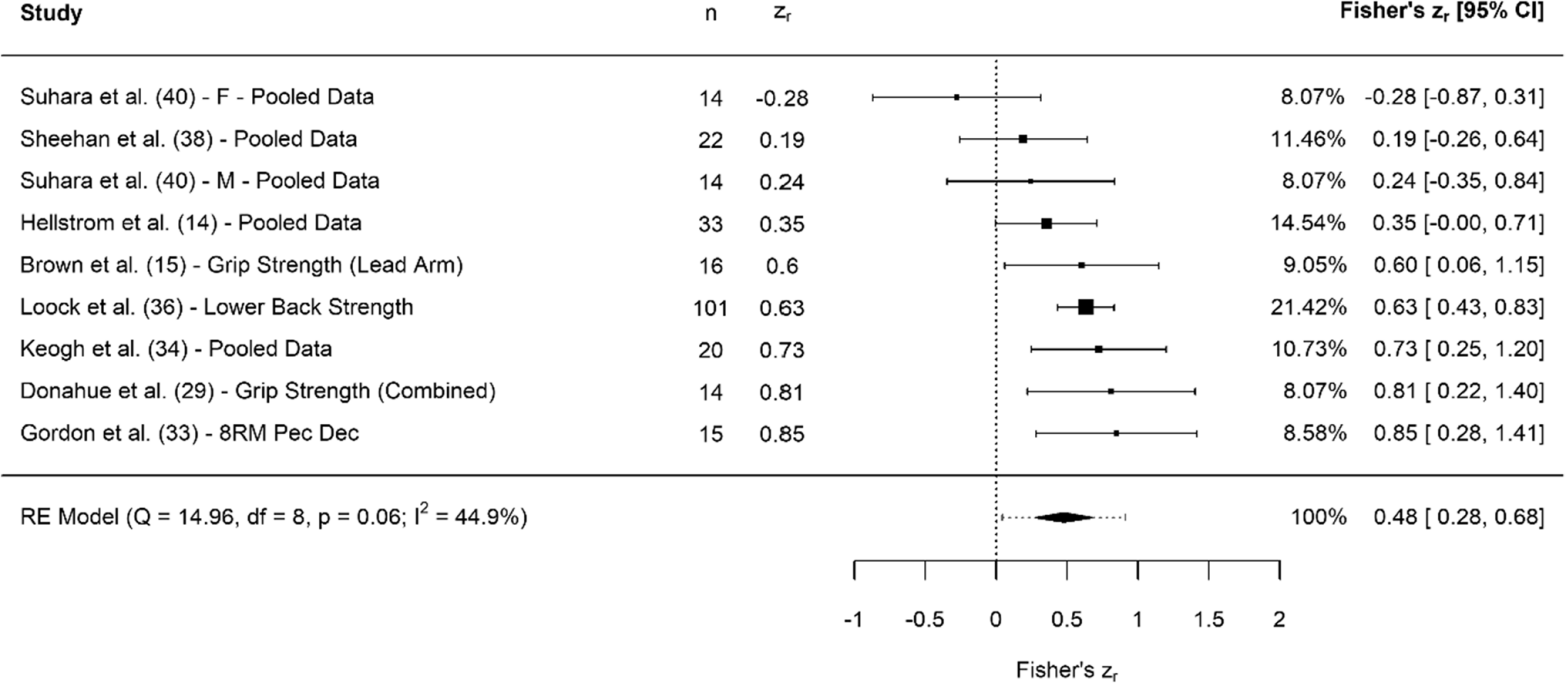
Forest plot showing the association (Fisher’s *z*_*r*_) between upper body strength and clubhead speed. *n* = sample size. *CI* confidence interval, *F* females, *M* males, *Pec Dec* resistance machine primarily involving use of the pectoralis major muscles, *RE* random effects, *RM* repetition maximum

**Fig. 4:**
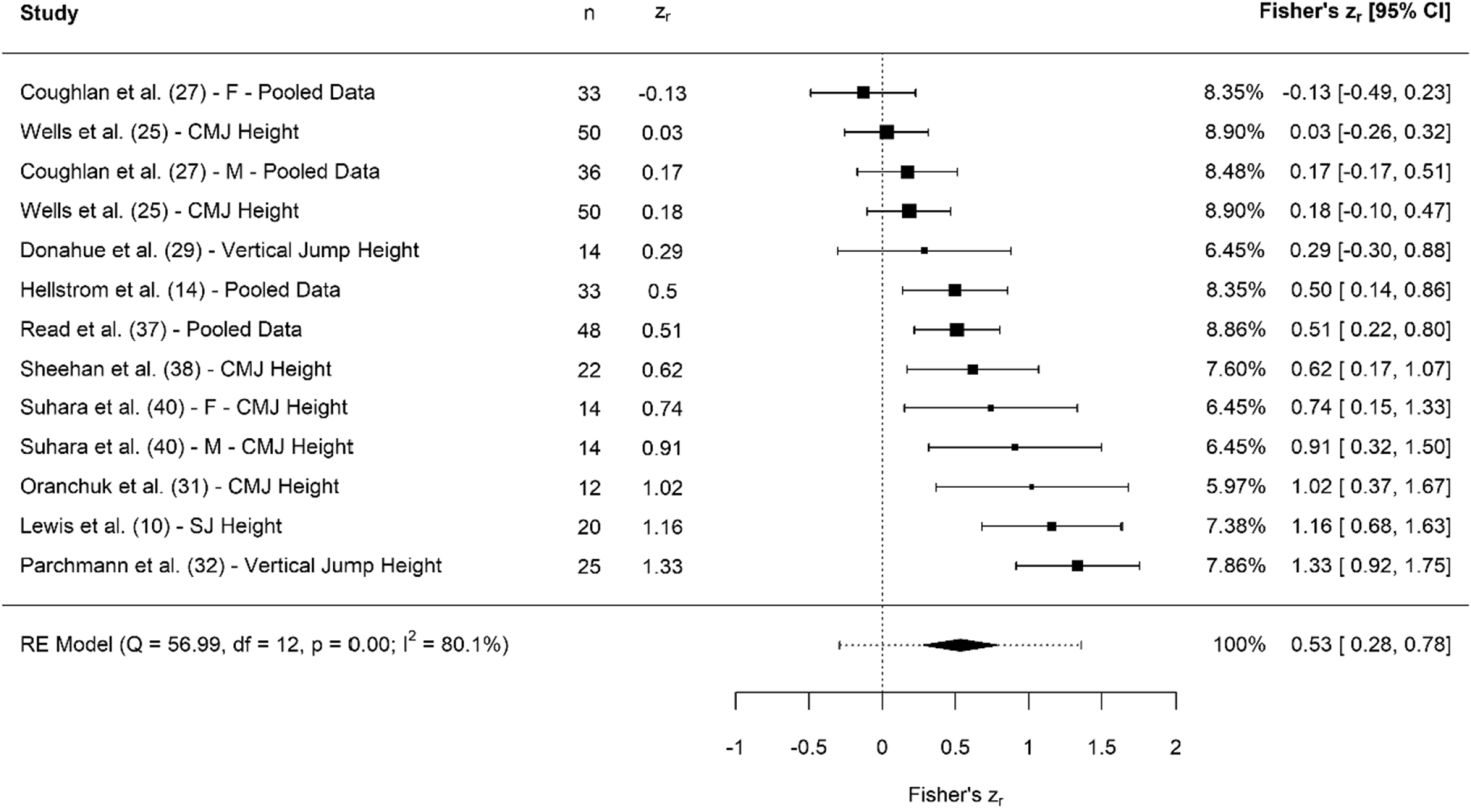
Forest plot showing the association (Fisher’s *z*_*r*_) between jump displacement and clubhead speed. *n* = sample size. *CI* confidence interval, *CMJ* countermovement jump, *F* females, *M* males, *RE* random effects, *SJ* squat jump

**Fig. 5:**
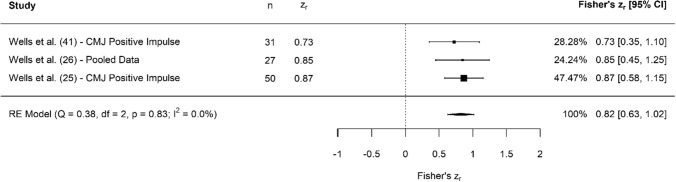
Forest plot showing the association (Fisher’s *z*_*r*_) between jump impulse and clubhead speed. *n* = sample size. *CI* confidence interval, *CMJ* countermovement jump, *RE* random effects

**Fig. 6:**
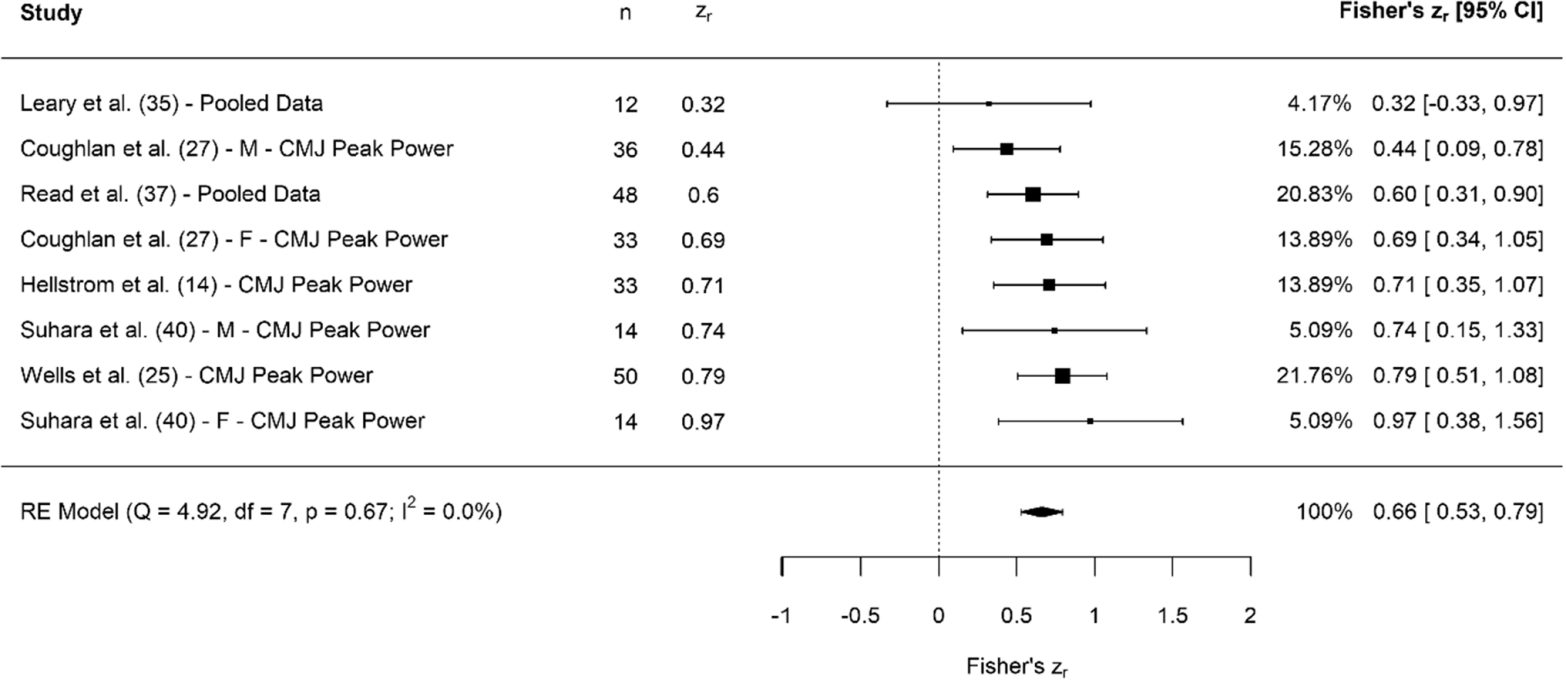
Forest plot showing the association (Fisher’s *z*_*r*_) between jumping peak power and clubhead speed. *n* = sample size. *CI* confidence interval, *CMJ* countermovement jump, *F* females, *M* males, *RE* random effects

**Fig. 7:**
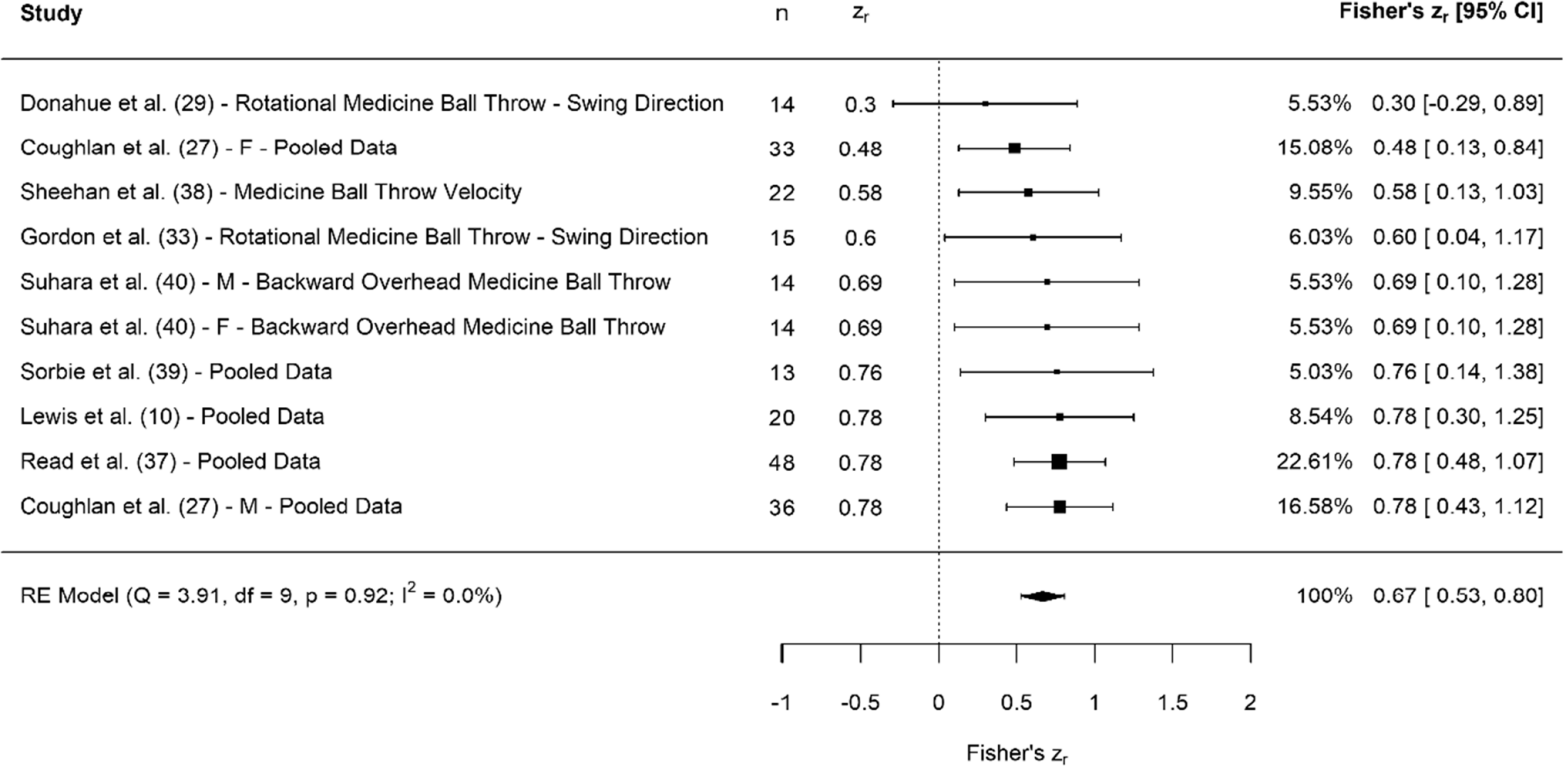
Forest plot showing the association (Fisher’s *z*_*r*_) between upper body explosive strength and clubhead speed. *n* = sample size. *CI* confidence interval, *F* females, *M* males, *RE* random effects

**Fig. 8:**
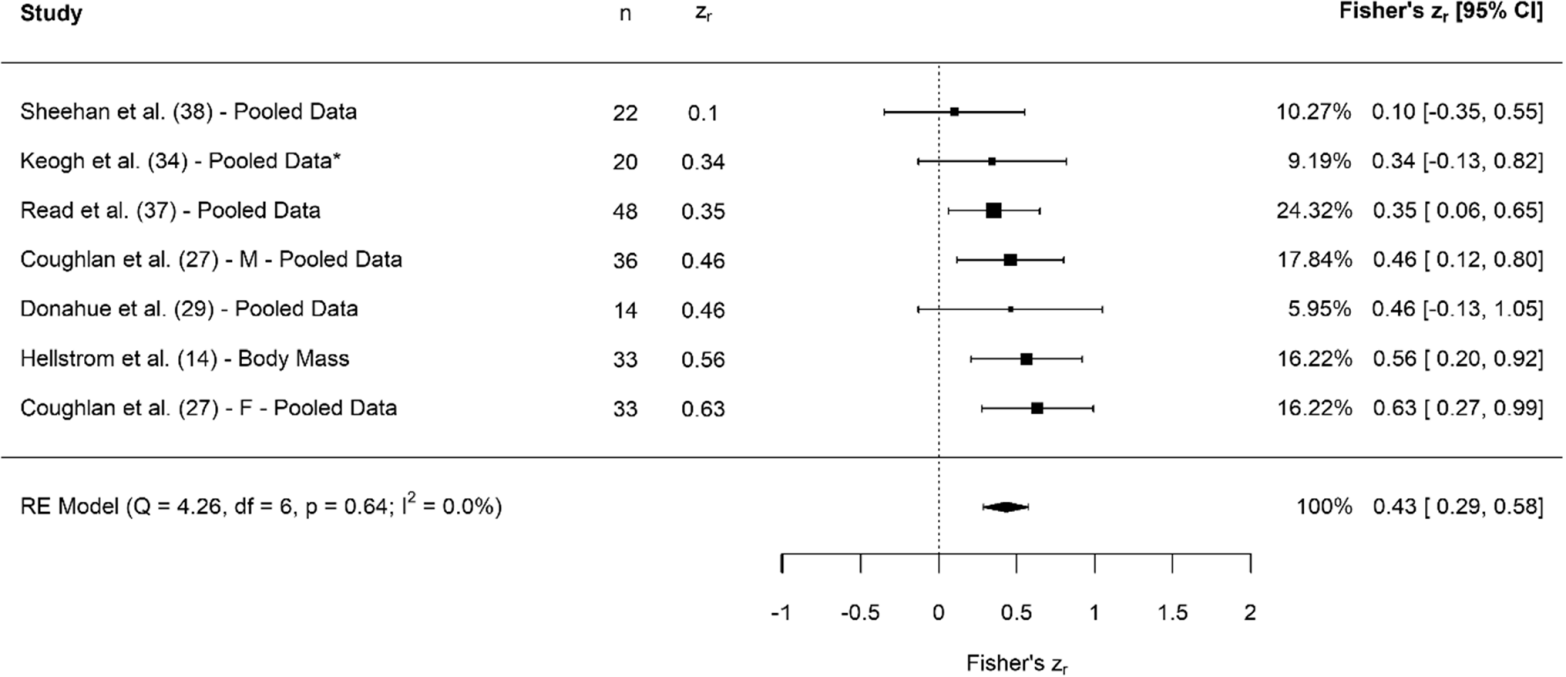
Forest plot showing the association (Fisher’s *z*_*r*_) between anthropometric measures and clubhead speed. *n* = sample size; *Denotes that directional differences existed in individual correlations, resulting in negatively aligned data being multiplied by minus 1, to directionally align all data prior to pooling. *CI* confidence interval, *F* females, *M* males, *RE* random effects

**Fig. 9:**
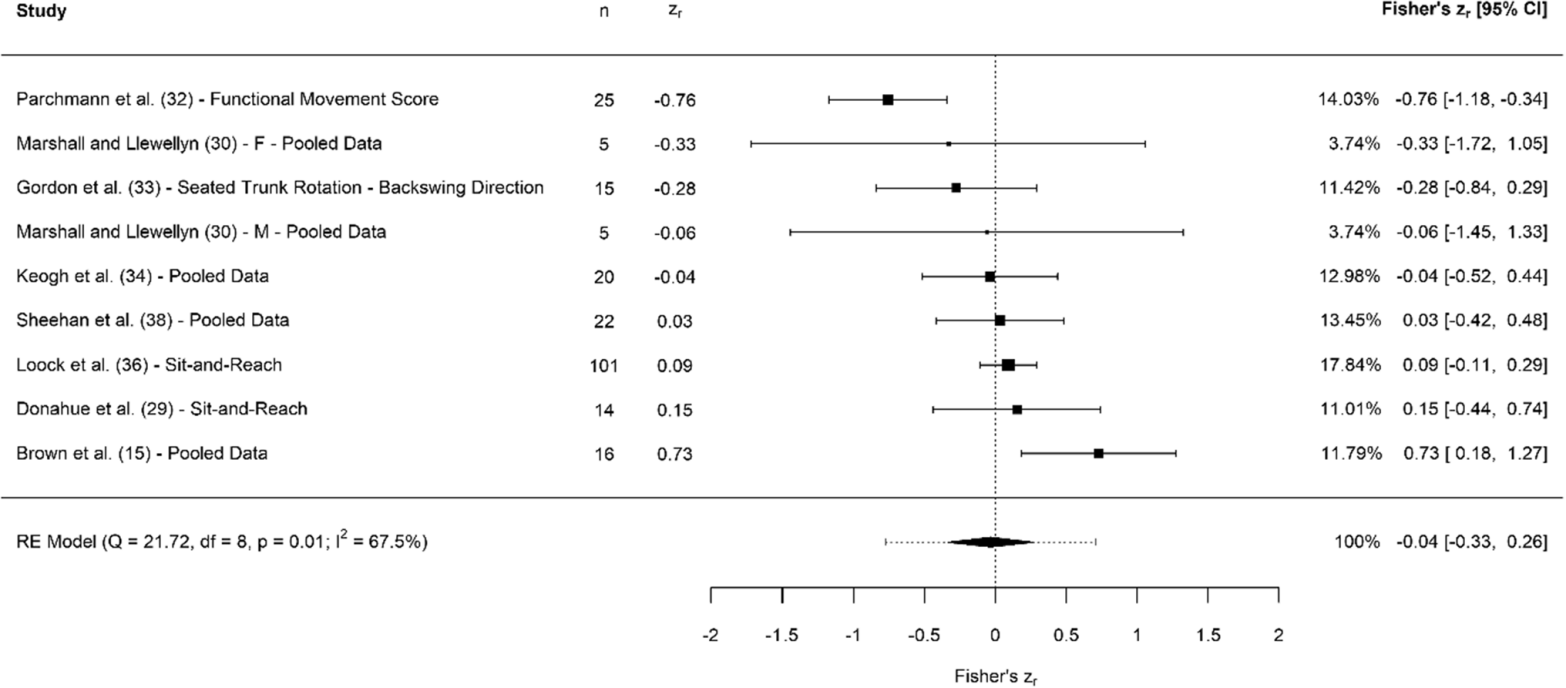
Forest plot showing the association (Fisher’s *z*_*r*_) between flexibility measures and clubhead speed. *n* = sample size. *CI* confidence interval, *F* females, *M* males, *RE* random effects

**Fig. 10:**
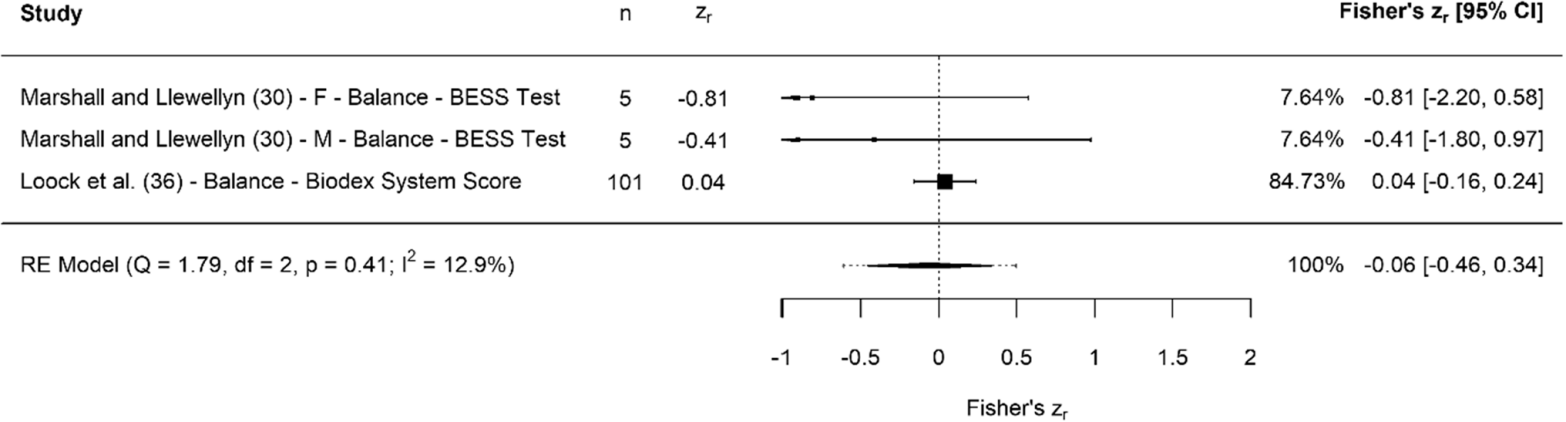
Forest plot showing the association (Fisher’s *z*_*r*_) between balance and clubhead speed. *n* = sample size. *BESS* Balance Error Scoring System, *CI* confidence interval, *F* females, *M* males, *RE* random effects

**Fig. 11:**
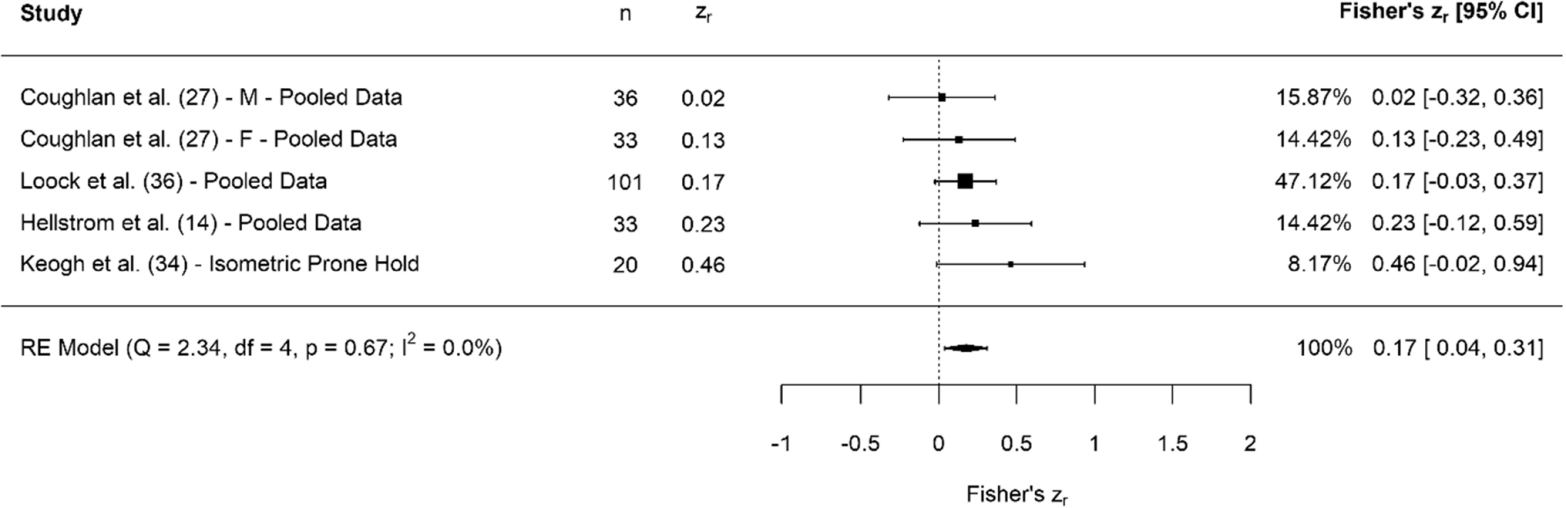
Forest plot showing the association (Fisher’s *z*_*r*_) between measures of muscle capacity and clubhead speed. *n* = sample size. *CI* confidence interval, *F* females, *M* males, *RE* random effects

#### Lower Body Strength

Collectively, results showed that lower body strength had small, significant associations with CHS (*z*_*r*_ = 0.47 [95% confidence interval {CI} 0.24–0.69], *r* = 0.44 [95% CI 0.24–0.60], *Z* = 4.03, *p* < 0.001). Tests for heterogeneity were identified as significant and moderate (*I*^2^ = 51.09%, *Q* = 18.20, *p* = 0.03), and there was no significant evidence of small study bias (z = − 0.758, *p* = 0.45). Lower body strength assessments included 1RM back squat [14, 31, 32], isometric mid-thigh pull (IMTP) peak force [26, 28, 41], isometric squat peak force [38], hack squat estimated 1RM [34], 1RM deadlift [31], 1RM clean [31] and hip extension and flexion strength [40].

#### Upper Body Strength

Collectively, results showed that upper body strength had small, significant associations with CHS (*z*_*r*_ = 0.48 [95% CI 0.28–0.68], *r* = 0.45 [95% CI 0.27–0.59], *Z* = 4.67, *p* < 0.001). Tests for heterogeneity were identified as low (*I*^2^ = 44.89%, *Q* = 14.96, *p* = 0.06), and there was no evidence of small study bias (z =  − 0.51, *p* = 0.61). Assessments of upper body strength included grip strength [14, 15, 29, 38], lower back strength [36], torso rotational strength [40], concentric-only push-up peak force [38], 1RM bench press [34], pec-dec (resistance machine primarily involving use of the pectoralis major muscles) 8RM [33], bar dips (repetitions × body weight) [14], pull-ups (repetitions × body weight) [14] and golf swing cable chop [34].

#### Lower Body Explosive Strength: Jump Displacement

Collectively, results showed that jump displacement had moderate, significant associations with CHS (*z*_*r*_ = 0.53 [95% CI 0.28–0.78], *r* = 0.49 [95% CI 0.27–0.65], *Z* = 4.17, *p* < 0.001); however, significant and high heterogeneity was noted (*I*^2^ = 80.09%, *Q* = 56.99, *p* < 0.001), along with significance regarding small study bias (*z* = 2.22, *p* = 0.03); however, the model utilised estimated no missing studies based on the trim and fill method. Jump displacement was assessed in three different tests, which were the CMJ [14, 27, 31, 32, 37, 38, 40], squat jump [10, 14, 37] and broad jump [27].

#### Lower Body Explosive Strength: Jump Impulse

Collectively, results showed that jump impulse (which was recorded across the CMJ, drop jump and squat jump) had large, significant associations with CHS (*z*_*r*_ = 0.82 [95% CI 0.63–1.02], *r* = 0.68 [95% CI 0.56–0.77], *Z* = 8.18, *p* < 0.001). Tests for heterogeneity were identified as trivial (*I*^2^ = 0.00%, *Q* = 0.38, *p* = 0.83), and there was no evidence of small study bias (z = − 0.32, *p* = 0.75). Positive impulse was measured in three different jump assessments: the CMJ [25, 26, 41], squat jump [26] and drop jump [26].

#### Lower Body Explosive Strength: Jump Peak Power

Collectively, results showed that peak power during jumping had moderate, significant associations with CHS (*z*_*r*_ = 0.66 [95% CI 0.53–0.79], *r* = 0.58 [95% CI 0.49–0.66], *Z* = 9.70, *p* < 0.001). Tests for heterogeneity were identified as trivial (*I*^2^ = 0.00%, *Q* = 4.92, *p* = 0.67), and there was no evidence of small study bias (*z* = − 0.03, *p* = 0.97). Peak power was measured in two jump assessments, which were the CMJ [14, 25, 27, 35, 37, 40] and squat jump (*n* = 1) [37].

#### Upper Body Explosive Strength

Collectively, results showed that upper body explosive strength had moderate, significant associations with CHS (*z*_*r*_ = 0.67 [95% CI 0.53–0.80], *r* = 0.58 [95% CI 0.49–0.66], *Z* = 9.39, *p* < 0.001). Tests for heterogeneity were identified as trivial (*I*^2^ = 0.00%, *Q* = 3.91, *p* = 0.92), and there was no evidence of small study bias (*z* = − 0.63, *p* = 0.53). Explosive strength was assessed in a number of assessments, including seated medicine ball throws [10, 27, 35], rotational medicine ball throws [10, 27, 33, 37], medicine ball throw velocity [38], ballistic bench press [39] and the backward overhead medicine ball (BOMB) throw [40].

#### Anthropometrics

Collectively, results showed that measures of anthropometry had small, significant associations with CHS (*z*_*r*_ = 0.43 [95% CI 0.29–0.58], *r* = 0.41 [95% CI 0.28–0.52], *Z* = 5.86, *p* < 0.001). Tests for heterogeneity were identified as trivial (*I*^2^ = 0.00%, *Q* = 4.26, *p* = 0.64), and there was no evidence of small study bias (*z* = − 0.44, *p* = 0.66). A number of anthropometric measures were utilised, including body mass [14, 27, 34, 37, 38], height [27, 29, 37–39], body mass index [34, 38], body fat % [34], limb length [34, 37], sum of skinfolds [34], fat mass [34] and fat-free mass [34].

#### Flexibility

Collectively, results showed that measures of flexibility had trivial, non-significant associations with CHS (*z*_*r*_ = − 0.04 [95% CI − 0.33 to 0.26], *r* = − 0.04 [95% CI − 0.32 to 0.25], *Z* = − 0.23, *p* = 0.82). Tests for heterogeneity were identified as high (*I*^2^ = 67.53%, *Q* = 21.72, *p* = 0.006), and there was no evidence of small study bias (*z* = − 0.13, *p* = 0.89). Flexibility was measured through several individual assessments, including the functional movement screen tests [32], sit-and-reach test [30, 36, 38], seated trunk rotation [15, 33, 38], standing trunk flexibility [15] and hip internal and external rotation [38].

#### Balance

Collectively, results showed that measures of balance had trivial, non-significant associations with CHS (*z*_*r*_ = − 0.06 [95% CI − 0.46 to 0.34], *r* = − 0.06 [95% CI − 0.43 to 0.33], *Z* = − 0.29, *p* = 0.77). Tests for heterogeneity were identified as trivial (*I*^2^ = 12.95%, *Q* = 1.79, *p* = 0.41), and there was no evidence of small study bias (*z* = − 1.28, *p* = 0.20). Balance was measured through two distinct methods, the Balance Error Scoring System (BESS) test [30] and the Biodex System Score [36].

#### Muscle Capacity

Collectively, results showed that measures of muscle capacity had trivial, significant associations with CHS (*z*_*r*_ = 0.17 [95% CI 0.04–0.31], *r* = 0.17 [95% CI 0.04–0.30], *Z* = 2.51, *p* = 0.01). Tests for heterogeneity were identified as trivial (*I*^2^ = 0.00%, *Q* = 2.34, *p* = 0.67), and there was no evidence of small study bias (*z* = 0.52, *p* = 0.60). Capacity was measured via maximum repetitions during push-ups [27, 36], pull-ups [14, 27], dips [14], sit-ups [14, 36], isometric prone hold [34] and wall squats [36].

## Discussion

This systematic review with meta-analysis set out to determine the associations between physical characteristics and CHS in golfers. Significant small to large pooled associations were evident between CHS and lower body strength, upper body strength, a range of jump variables, upper body explosive strength, and measures of anthropometry. Interestingly, trivial non-significant associations were evident between CHS and measures of flexibility and balance. The forthcoming sub-sections will address these associations in detail, for each physical characteristic. However, it should be noted that significant heterogeneity was evident for (1) lower body strength, (2) jump displacement and (3) flexibility, indicating that some level of caution should be applied when generalising our findings for these physical characteristics to a wider golf population.

### Lower Body Strength

Collectively, small correlations were reported between lower body strength and CHS (Fig. 2). These findings are in partial agreement with a review by Ehlert [13], who reported weak to strong correlations with CHS (*r* = 0.27–0.66). Despite this relationship being small, we maintain that strength is a key physical attribute for golfers to develop [12]. First, a golfer who possesses strength in the lower extremities is likely to have a more ‘stable base’, which probably allows for a more effective transfer of force both through the kinetic chain [2, 38] and between limbs during the inevitable changes in centre of pressure [42]. Previous research has suggested a golfer’s ability to efficiently produce and transfer force up through their body is advantageous for producing high CHS [43]. Second, the duration of the downswing typically lasts < 0.30 s [44, 45]. However, an essential component for golfers is the ability to separate the hips and the thoracic spine during the backswing (commonly termed the X-factor stretch), which results in large spikes in vertical ground reaction force before the downswing is initiated, coupled with changes in centre of pressure towards the lead leg [46]. Consequently, this conscious increase in force production is likely to be closer to 0.4–0.5 s before impact occurs, which is not dissimilar to the time required to produce maximal force [47]. This theory is partly supported by the current findings of this review and previous cross-sectional studies, where highly skilled golfers possessed greater levels of strength than their less skilled counterparts [34, 48].

Most commonly, squat variations were utilised as assessments of lower body strength [14, 29, 31, 32]. Despite 1RM free-weight testing being a commonly used assessment method for assessing lower body maximal strength, the hack squat demonstrated similar associations with CHS, and therefore may be a useful tool for practitioners who have access to the required equipment and are working with golfers who have limited resistance training experience. Further studies utilised the use of force plates to perform IMTP assessments presenting associations between peak force and CHS [26, 28, 41]. When force plates are a viable option, IMTP assessments can provide an accurate assessment of maximal force production in the lower body, which has been highlighted as having positive associations with CHS in European Tour golfers [41]. Ideally, when assessing lower body strength, multi-joint protocols (e.g. IMTP, 1–3RM back squat) should be prioritised over single-joint methods (e.g. isokinetic dynamometry), because they better (although not perfectly) represent the movement demands of a golfer [2, 38].

### Upper Body Strength

Upper body strength showed small associations with CHS (Fig. 3). Similar to the aforementioned narrative for lower body strength, upper body strength should not be ignored. First, and potentially most importantly, if upper body strength is improved, it will increase the ability for relevant tissues to withstand load and may logically reduce the likelihood of injury. In turn, this may assist in maximising availability for practice and competition, ensuring golfers are able to continually swing at high speeds [49]. This suggestion is part of the Probability of Performance Model created by Brearley et al. [49], which indicates the most influential impact of physical training is its ability to maximise a player’s availability to practice and compete. Second, strength is the foundation for optimising speed and power development [50], and pooled associations between CHS and upper body explosive strength were noticeably larger than for upper body strength (discussed in Sect. 4.4).

Several assessments were employed to measure upper body strength, with grip strength being the most prominent [14, 15, 29, 38]. This finding is consistent with previous research, showing an association between lead side grip strength and golf performance. Komi et al. [51] reported that the lead hand exerts the most pressure during the downswing phase to control the swing, helping to transfer to greater CHS. Three assessments utilised multi-joint exercises for upper body strength (bench press, pec-dec machine and golf swing cable chop), also reporting positive associations with CHS. However, the pec-dec machine operates in a single plane of motion (which is not representative of the movements in golf), and the cable chop, whilst a useful supplementary exercise for golfers, is not an appropriate measure of true upper body strength. Thus, multi-joint assessments such as the bench press that assess a percentage of RM or velocity loss (if using appropriate technology) are recommended.

### Lower Body Explosive Strength: Jump Displacement, Impulse and Peak Power

Despite these appearing as separate metrics in the results, we have chosen to discuss these together, given that impulse is the key determinant for how high or far someone can jump [52]. In addition, one study utilised the standing long jump as an assessment method [27], resulting in the terminology ‘jump displacement’ rather than ‘jump height’ being used. Several studies assessed the association between jump height and CHS, with moderate associations evident (Fig. 4). Despite jump height being the most typical outcome measure reported from jump testing (and the moderate associations reported in this review), we argue that it may not be the most appropriate metric to report for golfers. First, with CHS being a critical performance indicator in golf, this can be positively affected by an increase in body mass. However, increases in body mass may also be counter-productive to concurrently trying to improve jump height. Second, with additional body mass likely being of greater importance to golf than the ability to jump high, a better jump variable is likely to be ‘jump momentum’ (mass × velocity). This is because CHS is driven by angular momentum, which body mass contributes to [25, 26]; thus, the ability to generate momentum is likely to be a better proxy measurement of CHS than jump height, which is limited by body mass. Furthermore, in an ideal scenario, practitioners should evaluate jump performance using force plates, enabling a more detailed insight into both outcome measures and jump strategy [53]. However, even if practitioners only have access to a jump mat or a smartphone application, Wells et al. [25] showed how inverse dynamics can be used to quantify net impulse, when the only metrics available are jump height and a player’s body mass. Thus, with momentum being a key metric in golf, and the change in momentum being directly proportional to the impulse generated during a jump, monitoring impulse (or momentum) should be prioritised over the commonly reported outcome measure of jump height, which is supported by the initial large associations noted with CHS (Fig. 5).

Similar to jump height, pooled associations for peak power showed moderate associations with CHS (Fig. 6). Practically speaking, with power being a representation of force × velocity, and with the importance of force already discussed, it stands to reason that peak power is a useful metric to monitor as well. Put simply, the more force that can be applied during the high-velocity nature of a swing will result in increases in power production. This is also supported by Nesbit and Serrano [54], who noted that a corresponding decrease in both peak power and CHS were present with an increase in handicap (i.e. lesser skilled golfers). However, it should be acknowledged that lesser skilled golfers may possess similar force capabilities but may be less able to transfer this to their swing. Furthermore, when viewing Fig. 6, only one study [35] exhibited a CI that crossed zero, highlighting the consistent nature of peak power during jumping and its association with CHS.

### Upper Body Explosive Strength

Our data showed moderate associations between measures of upper body explosive strength and CHS (Fig. 7). As previously discussed, the upper body is likely to have less time to produce force than the lower body. Consequently, ballistic force production or explosive strength may be more important for the upper body (compared to strength), and our data support this suggestion, given the considerably higher pooled associations with CHS. These findings are in agreement with Hume et al. [2], who suggested that muscles of the upper extremities are highly active during the swing to produce a powerful stretch–shortening cycle activity [2]. Specifically, the pectoralis major has been reported as the most active muscle of the upper body (when reported via electromyography as a percentage of maximal voluntary isometric contraction) during the early (64% on the trail side) and late downswing (93% trail and lead side) phases [55]. With this in mind, it seems prudent to suggest that upper body ballistic exercises, which require rapid activation of the chest muscles, would form appropriate assessments for physical profiling golfers. The most common methods utilised in the research to date are various medicine ball throws, most likely because they are easy to implement and provide a quick outcome measure of distance thrown. Whilst this method is not being down played, some of these assessments were done standing and would include major contributions from the lower body (which cannot be fully quantified). Thus, if medicine ball throws are considered the most viable option, it is suggested that these are done in a seated position, to isolate the assessment to the upper body. In addition, further research should consider alternative methods of assessment such as the bench press throw, given that it is also isolated to the upper body, is ballistic in nature and has strong involvement of the pectoral muscles.

### Anthropometry

Pooled associations showed that measures of anthropometry (e.g. height, body mass, limb length) had small associations with CHS (Fig. 8). However, the nature of how these data are reported needs careful consideration. For example, six out of seven studies had pooled data, indicating that more than one measurement was utilised during the Fisher’s *z* transformation. As such, it becomes challenging to truly determine which measure of anthropometry (e.g. height, body mass, limb length) has the greatest influence on CHS. Theoretically, it seems plausible to suggest that golfers with greater body mass will possess more muscle mass and, therefore, likely be able to produce more force during the golf swing. Aside from mass-related measures, all additional assessments of anthropometrics consisted of non-modifiable factors such as height and limb length. Although little can be done to change these non-modifiable factors, they should not be ignored. First, it should be noted that longer limbs may enable a longer and wider path length when swinging the club, which may have positive effects on rotational torque and power [2]. For example, MacKenzie et al. [56] reported that if hand path length was increased by 0.12 m, then CHS would likely increase by approximately 2.7 mph. Second, where physical training is concerned, longer limbs may have an impact on which exercises are selected for players. For example, achieving full-depth squats may be harder for some taller players, and in such cases, additional options such as box, pin or partial squats may be a viable alternative enabling the desired physical adaptation to still be achieved.

### Flexibility

Pooled correlations showed trivial findings between flexibility and CHS (Fig. 9), which is in agreement with previous literature [13]. These findings are perhaps some of the most interesting in this meta-analysis, given the commonly held belief amongst golfers and practitioners that stretching is an essential component of training. This is supported in a recent survey by Wells and Langdown [57], who reported that ‘flexibility and stretching’ was the most common training modality employed by highly skilled golfers (handicap = 0.42 ± 2.81) during the in-season. It is important to reiterate that our findings show there was a wide variety of assessments used, some of which seem highly irrelevant for golfers (e.g. sit-and-reach test). Therefore, the lack of important findings may be a consequence of poor ecological validity of some of the selected assessments, rather than a complete lack of importance of flexibility altogether. Furthermore, given the previously mentioned importance of separating the hips and the thoracic spine, it should be acknowledged that if a golfer is unable to do that sufficiently, it is likely that they will simply find an alternative movement strategy to achieve the outcome they need during the swing [58]. Such examples may include greater internal rotation of the hip or knee bend on the lead leg during the back swing, enabling the desired level of rotation prior to the start of the downswing. Consequently, it is suggested that more appropriate measures of flexibility are chosen, such as seated thoracic rotation, enabling the upper body (i.e. thoracic spine region) to be isolated, which has been suggested in recent golf literature [12]. Additionally, and as a final point of consideration, it seems plausible to suggest that flexibility may be more bespoke in its application for golfers than other physical characteristics, given it is likely to be based on a player’s preferred movement approach to a given task – namely, the swing. Put simply, increasing flexibility may open up a wider variety of shot options for some players who need improvements, whilst for others it may not be necessary. In contrast though, and given the results in Figs. 5, 6 and 7, it is hard to see how any golfer would not benefit from the ability to enhance force production ballistically.

### Balance

Pooled correlations showed trivial findings between balance and CHS (Fig. 10), inclusive of only two studies [30, 36], which showed noticeably different results. First, Marshall and Llewelyn [30] conducted an associative analysis with sample sizes of five (for both males and females), which is undoubtedly too small to provide any meaningful conclusions as to the link between balance and CHS. In contrast, and although employing a different balance assessment, Loock et al. [36] evaluated a sample of 101 golfers, accounting for nearly 85% of the weighting in our meta-analytical statistics for this physical characteristic. This provides considerably stronger evidence concerning the link between balance and CHS, which appears to be negligible. Intuitively as well, despite shifts in centre of pressure during the swing [42], golfers are very rarely off-balance and never take a shot standing on one leg. Thus, it becomes challenging to suggest that balance exercises or assessment protocols should form a staple part of a golfer's training or testing practices.

### Muscle Capacity

Pooled correlations showed trivial, but significant, associations between measures of muscle capacity and CHS (Fig. 11). First, it is important to outline that measures of muscle capacity were completed in bodyweight assessments, consisted of both upper and lower body protocols, and utilized outcome measures of either maximal repetitions or maximal time. Despite these data being significant, they highlight a very important distinction when compared with data for lower body or upper body strength. As the results show, associations were noticeably lower than for lower and upper body strength, which we believe to be an accurate representation of the physical requirements for golf. For example, despite the swing being a highly repetitive action, long periods of rest are provided between shots during competition, enabling sufficient recovery before the next shot is taken. Furthermore, some elements of the game are underpinned almost exclusively by skill (e.g. putting and chipping), with limited requirements for physical capacity. Thus, it stands to reason that maximal and explosive force production are of greater importance than muscle capacity or endurance, especially when considering maximal effort shots (e.g. using a driver, wood or long iron). With this in mind, practitioners should not only consider whether muscle capacity assessments offer any real benefits that support decision-making in golf, but also whether training methods that develop this muscular adaptation are truly needed. Although somewhat anecdotal, we believe that training programmes in the gym should be more focused on maximal strength and ballistic force production, with typically lower repetitions (i.e. ≤ 6) than are often achieved in these muscle capacity assessments.

### Summary

**Fig. 12:**
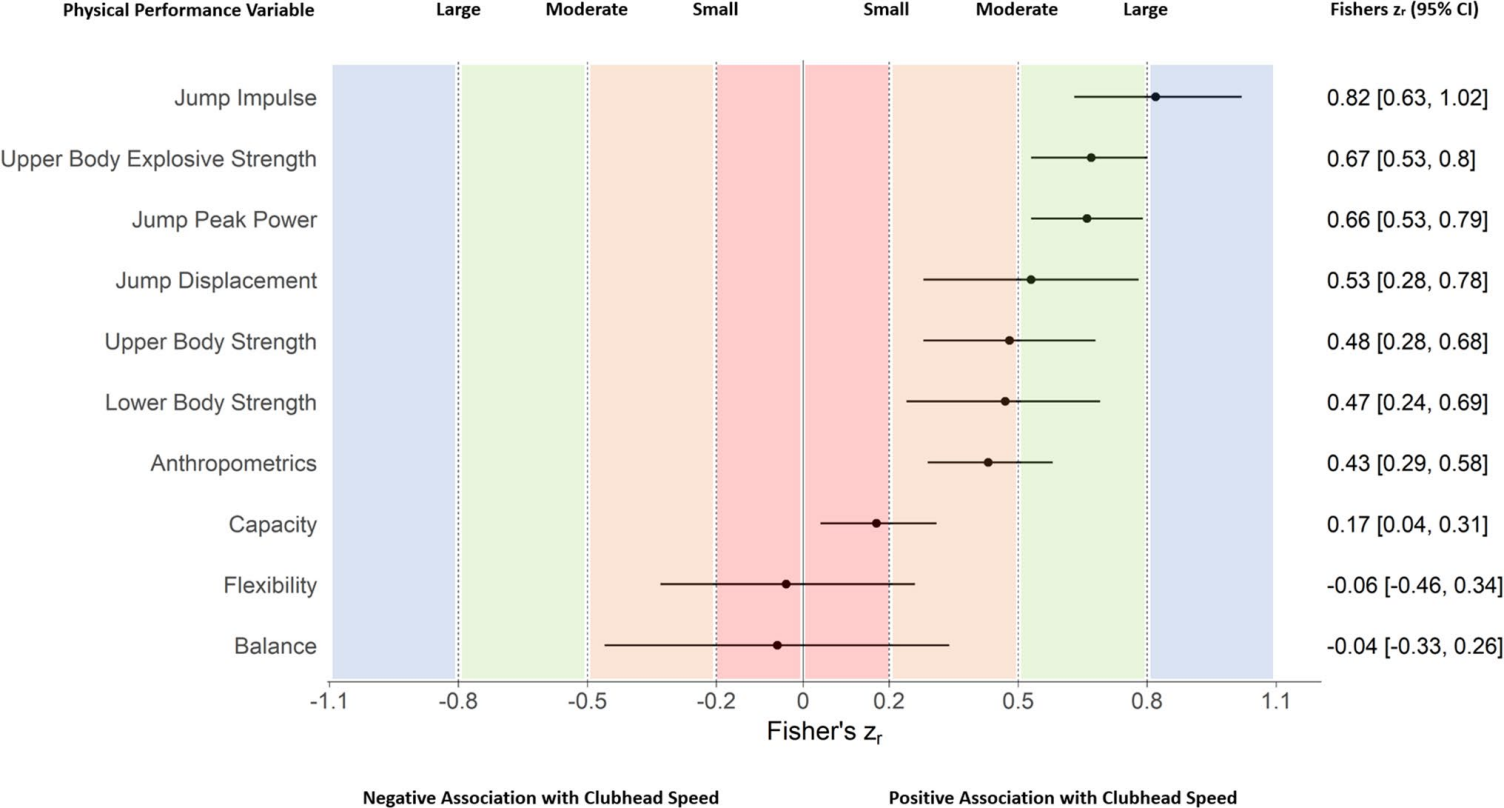
Summary forest plot showing the association (Fisher’s *z*_*r*_) between different physical characteristics and golf clubhead speed. *CI* confidence interval

Given the magnitude of data reported and discussed in this review, we have provided a summary plot reporting the mean summary effect estimates for all physical characteristics and their association with CHS (Fig. 12).

### Limitations and Directions for Future Research

The findings of this current review should be analysed with the acknowledgement of a few limitations, which in turn provide useful guidance for future research suggestions. First, several protocols were often selected for any given physical characteristic. For example, lower body strength was measured using a 1RM back squat, hack squat, IMTP and even isokinetic dynamometry. Although all provide some measure of lower body strength, they do not measure the same thing, and where possible, greater consistency should be utilised between assessment protocols and studies, with our aforementioned suggestions throughout the discussion, for each physical characteristic, considered for the sport of golf. Specifically, dynamic assessments of lower body strength (e.g. 1RM back squat) appear to show stronger associations with CHS than isometric assessments (e.g. IMTP). Thus, RM-based protocols maybe favourable when assessing golfers, although it should be acknowledged that such methods require substantial time investment, which may not always be possible. For upper body strength, there was a distinct lack of consistent protocols between the included studies. However, in line with suggestions for lower body strength, RM-based protocols for the bench press may be useful given the dynamic nature of the assessment and primary focus on the pectoralis muscle group. Assessments that measure explosive force production should be prioritised given the noticeably larger associations with CHS reported in this review, and can be achieved using relatively simple methods. For example, jump testing on force plates is now common in sport science, which will enable the quantification of net impulse (noting that this is the metric with the strongest association with CHS). However, even if practitioners only have access to a jump mat or smartphone application, inverse dynamics can be used to convert jump height into a net impulse value [41]. In addition, medicine ball throws serve as a simple and useful assessment of explosive force production capability in the upper body. Finally, and importantly for those working in golf, many studies using measures of flexibility to date were questionable. If it is deemed that this physical characteristic should be assessed, it is suggested that practitioners consider using the seated thoracic rotation assessment as a means of quantifying ‘flexibility’ [12]. This method provides an assessment of rotational mobility (as required in golf), which also serves to separate movement between the hips and thoracic spine, a concept known to be critical for the development of CHS [46].

Second, very few studies included have been conducted in female golfers, and this huge disparity between sexes is evident in a recent meta-analysis by Robinson et al. [58], which reported only three associative studies and six training interventions assessing the associations between and effects of physical training on CHS. Thus, future research should aim to address this imbalance. Third, it is very rare to see individual data analysis in golf studies [59], which is a surprise given the individual nature of the sport. Moving forward, we suggest that practitioners consider quantifying ‘true change’ in physical performance individually, by establishing whether differences are greater or less than the player’s associated test variability score. Specifically, when multiple trials of a given test are conducted, the standard deviation can be used to determine the natural bandwidth of variability for that athlete. Thus, and assuming the test in question exhibits acceptable levels of reliability, the standard deviation becomes the ‘target score’ by which practitioners can assess whether change is real [12]. Finally, future studies may wish to consider utilising some shot-related metrics beyond CHS and branch into additional skill parameters such as strokes gained. Brennan et al. [11] proposed a framework for monitoring golf performance measures that link to strokes gained off the tee. More specifically, it is common for players and coaches to use launch monitor technology (e.g. Trackman, Flightscope) to quantify shot performance during practice. Many of these use radar technology, which is what is used when tracking missiles. Put simply, the technology is looking for an object flying through the air, so it seems more logical that metrics such as ball speed and carry distance are monitored, potentially over CHS, which has been previously suggested [60].

## Conclusion

In summary, our findings showed trivial to large associations between physical characteristics and CHS. More specifically, measures of explosive force production appear to be more important than maximal strength measures in both the lower and upper body. Metrics such as impulse and peak power are useful proxy measures from jump testing, and medicine ball throws serve as a practical method of assessing explosive force production in the upper body. In contrast, the association between flexibility/balance and CHS appears to be minimal (noting that the quality of evidence for these two physical characteristics is questionable). Practitioners can use these findings to better establish more appropriate physical tests that relate to CHS, which in turn can be used to provide appropriate benchmark data when profiling a golfer’s physical capacities.

## Declarations

### Data availability

The data within this systematic review and meta-analysis are secondary data and available through the relevant articles referenced throughout. All statistical analyses were carried out using the ‘metafor’ package in R.
